# Supplementary material for: Competitive Coherence Generates Qualia in Bacteria and Other Living Systems
Source: Biology (Basel). 2021 Oct 12;10(10):1034. doi: 10.3390/biology10101034 (PMC8533353; doi:10.3390/biology10101034)
Supplement: Supplementary file 1 [file biology-10-01034-s001.zip › biology-1401159-supplementary.pdf]

CocoSOC3 Source code

'%%%%%%%%%%%%%%%%%%%%%%%%%%%%%%%%%%%%%% V E R S I O N D U P R O G R A M M E %%%

' %

' 3 December 2020 %

' %

'%%%
%%%
%%%%%%%%%%%%%%%%%%%%%%%%%%%%%%

Option Explicit ' VB avertira quand un nom de variable créé dans le code ne figure pas dans les déclarations ci-dessous

Option Base 1 ' Affecte la valeur 1 au plus bas niveau d'indice d'un tableau. La seule autre valeur possible est 0

'CHOICES:

'Emergence and compatibility do not operate at present but would do if the values in these subroutines were changed from 1

Const Test = 0 'put to 0 to avoid preloading a winning pattern; REMEMBER to alter testcompatibilitysub in Initialize

Const MetaLoop = 1 'Allows the program to run for a long time if greater than one

Const NowNextWeighting = 1 'Changes the relative importance of the Now and Next connections when filling the Activity Register

'Const CyclePermission = 1 '=0 for no cycling and = 1 for cycling - now a variable depending on success/failure

Const CycleLength = 1000 'typically set to 4 to cause cycling every 4 lines of Activity Register

Const ForcedOutputProbability = 2 'OutputLacking goes up by 1 for each line where there is no output (it is reset by detection of output)

'if the counter, OutputLacking, > ForcedOutputProbability Then OutputNeeded

Const NoiseLevel = 0 'for noise to cause insertion of a random element into the ActivityRegister,

'NoiseLevel has to be greater than R50 which is random between 1 and 20 (hence NoiseLevel has to be greater than 0

Const MutationThreshold = 90 'mutation threshold is only usedin the PunishMutate routines, range is from 1 to 100,

'high means few mutations are inserted during punishing MutationNow Int((100 * Rnd) + 1) < MutationThreshold then no punishing

```
Const ActivatePunish = 1 'If ActivatePunish = 0 then the punish routines are disabled

Const ActivateReward = 1 'If ActivateReward = 0 then the reward routines are disabled

Const KeepInputSameForThisNumber = 1 'Typically 1 for immediate cycling of inputs

Const NowTooBiasedLimit = 10 'typically = Knumber / 2

Const NextTooBiasedLimit = 10

Const RemoveInputByCoco = 0 'if there is a real input, a second artefactual one is removed

Const Enumber = 1000                    ' Ces 4 paramètres étant cités dans des Dim

Const Anumber = 4                  ' exemple :'Dim DownT1/ime (Enumber), il est impossible

Const Knumber = 30                 ' pour l'instant de les charger à partir de l'écran

Const DowntimeNumber = 16        ' Feuille principale

Const EndOfActivityRegister = 25000 'REMOVE and restore to 150

Const RepeatRewardPunish = 10

Const InputRange = 3

Const OutputRange = 3

Const InputTotalMax = 6

Const OutputRangeMax = 50

Const OutputRangeMin = 100


'END OF CHOICES



' %%%%%%%%%%%%%%%%%%%%%%%%%%%%%% Maurice - 05-06-2008 - Déclarations
pour séquences Maurice Affichages %%%%%%%%%%%%%%%%%%%%%%%%%

                        '%

Dim Spy, Esp, Esp1, xe, ye As Integer                 ' Pour diverses séquences Maurice
'%

'
%%%%%%%%%%%%%%%%%%%%%%%%%%%%%%%%%%%%%%%%%%%%%%%%
%%%%%%%%%%%%%%%%%%%%%%%%%%%%%%%%%%%%%%%%%%%%%%%%
%%%%%%%%%%%%%%%%%%%%%%%%%%%%%%

Dim Temp1 As Integer

Dim Temp2 As Integer

Dim Temp3 As Integer

Dim Temp4 As Integer
```

```
Dim Temp5 As Integer
Dim Temp6 As Integer
Dim Temp7 As Integer
Dim Temp8 As Integer
Dim Temp9 As Integer
Dim Temp10 As Integer
Dim Temp11 As Integer
Dim Temp12 As Integer
Dim Temp13 As Integer
Dim Temp14 As Integer
Dim Temp15 As Integer
Dim Temp16 As Integer
Dim Temp17 As Integer
Dim Temp18 As Integer
Dim Temp19 As Integer
Dim Temp20 As Integer

Dim ConnectivityNow(Enumber) As Integer
Dim ConnectivityNext(Enumber) As Integer
Dim HighestNextConn(Enumber, 2) As Integer
Dim TempHighestNextConn(2) As Integer
Dim TempHighestNowConn(2) As Integer
Dim HighestNowConn(Enumber, 2) As Integer

Dim BinNow(500) As Integer
Dim BinNext(500) As Integer
Dim BinnedScore As Integer
Dim ZeroNow As Integer
Dim ZeroNext As Integer

Dim RunWithoutInput As Integer
```

```
Dim ScrambleLowestNumber As Integer
Dim MarkScrambleLowestStart As Integer
Dim TempScrambleLowestAddress As Integer
Dim TempScrambleLowestScore As Integer

Dim UnusedElement(Enumber, 2) As Integer
Dim LowestUsedElement(Enumber, 2) As Integer
Dim TempUnusedElement(2) As Integer

Dim UsedElement(Enumber, 2) As Integer
Dim HighestUsedElement(Enumber, 2) As Integer
Dim TempUsedElement(2) As Integer

Dim SpareLink(Enumber, Enumber) As Integer
Dim LinkFrom As Integer
Dim LinkTo As Integer
Dim MatrixNow(Enumber, Enumber) As Integer
Dim MatrixNext(Enumber, Enumber) As Integer
Dim TempMatrixNow(Enumber, Enumber) As Integer
Dim TempMatrixNext(Enumber, Enumber) As Integer
Dim ValueOfMatrixNow As Integer
Dim ValueOfMatrixNext As Integer

Dim OutputFrequency(Enumber) As Integer
Dim LineChoice As Integer



Dim CitedElement As Integer
Dim CitingElement As Integer

Dim InputA(InputRange) As Integer
Dim OutputA(OutputRange) As Integer
```

```
Dim OutputNowScore(Enumber) As Integer

Dim OutputNeeded As Integer
Dim DontOverwriteNow As Integer
Dim DontOverwriteNext As Integer
Dim DontOverwriteNextOverlap As Integer
Dim CompatibilityTable(Enumber, Enumber) As Integer
Dim FirstElement As Integer
Dim SecondElement As Integer

Dim CounterForPreviousSuccess As Integer
Dim RewardDecision As Integer
Dim PositionAR1 As Integer
Dim PositionNow1 As Integer
Dim OtherPositionsAR1 As Integer
Dim PositionAR2 As Integer
Dim PositionNext1 As Integer
Dim OtherPositionsAR2 As Integer

Dim PositionAR3 As Integer
Dim PositionNext3 As Integer
Dim OtherPositionsAR3 As Integer

Dim PositionAR4 As Integer
Dim PositionNext4 As Integer
Dim OtherPositionsAR4 As Integer

Dim PositionAR5 As Integer
Dim PositionNext5 As Integer
```

```
Dim OutputLacking As Integer
Dim ForcedOutputPosition As Integer
Dim MutationFrequency As Integer
Dim MutationPosition As Integer
Dim MutationElement As Integer
Dim MutatedElement As Integer
Dim MutationNow As Integer
Dim MutationNext As Integer
Dim MutationOverlap As Integer

Dim Downtime(Enumber) As Integer
Dim ConstantInput As Integer
Dim InputTotal As Integer
'for Activity register need to indicate linenumber
Dim linenumber As Integer
Dim NewLineNumber As Integer
Dim Activity(EndOfActivityRegister, Anumber) As Integer
Dim Element(Enumber, 2 * Knumber) As Integer
Dim SignElement(Enumber, Enumber) As Integer
Dim Sign As Integer

'Calculate most frequent elements
Dim NextScoreRegister(Enumber) As Integer
Dim NowScoreRegister(Enumber) As Integer

Dim EmergentNextScoreRegister(Enumber) As Integer
Dim EmergentNowScoreRegister(Enumber) As Integer



Dim HighestNext(Enumber, 2) As Integer
Dim TempHighestNext(2) As Integer
```

```
Dim TempHighestNow(2) As Integer
Dim HighestNow(Enumber, 2) As Integer
'Dim TopNext As Integer
'Dim TopNow As Integer
Dim SavedHighestNext(Enumber, 2) As Integer
Dim SavedHighestNow(Enumber, 2) As Integer


Dim Bigloop0 As Long 'Byte
Dim Bigloop1 As Byte 'unused
Dim A As Integer
Dim E As Integer
Dim K As Integer 'for field within phase element
Dim L As Integer 'for field within phase element
Dim P As Integer 'for phase element
Dim Q As Integer 'for phase element
Dim S As Integer
Dim T As Integer
Dim RandomizeAgain As Integer
Dim InputTally As Integer
Dim JustTestingNow As Integer
Dim JustTestingNext1 As Integer
Dim JustTestingNext2 As Integer
Dim Filler As Integer


'Variables for using the Cycle routine
Dim CycleElement As Integer
Dim CycleStep As Integer
Dim Cycling As Integer
Dim CyclePermission As Integer
```

```
'Dim CycleLimit As Integer

'Variables for Scrambling Nows
Dim ScrambleNowNumber As Integer
Dim TempScrambleNowAddress As Integer
Dim TempScrambleNowScore As Integer
Dim MarkScrambleNowStart As Integer

'Variables for Scrambling Nexts
Dim ScrambleNextNumber As Integer
Dim TempScrambleNextAddress As Integer
Dim TempScrambleNextScore As Integer
Dim MarkScrambleNextStart As Integer

'Variables for Running Score (short term memory of successes)
Dim Adaptation As Integer
Dim UseAdaptation As Integer
Dim DeltaRunningScore As Integer
Dim TimeToUseRunningScore As Integer
Dim RunningScorePointer As Integer
Dim RunningScoreTotal1 As Integer
Dim RunningScoreTotal2 As Integer
Dim RunningScoreTotal As Integer
Dim RunningScore(20000) As Integer 'note that this was Enumber but crashes if Enumber is small
Dim RunningScoreAction As Integer
Dim IncrementRunningScore As Integer
Dim RunningScoreWindow As Integer
Dim RunningScoreLength As Integer
Dim SuccessTableWindow As Integer
Dim PresentResult As Integer
Dim SameResult As Integer
```

```
'Variables for neighbourhood connections
Dim MakeLocalConnection As Integer
Dim MakeDistalConnection As Integer
Dim TwoInputs As Integer
Dim MakeLocalNextConnection As Integer
Dim MakeDistalNextConnection As Integer

Dim MakeLocalNowConnection As Integer
Dim MakeDistalNowConnection As Integer
Dim MakeLocalOverlapConnection As Integer
Dim MakeDistalOverlapConnection As Integer

'Victor addition to Coco20atelier 7-6-2008
Dim ModuloRemainder As Integer
Dim WithinLocalNextRangeOutput As Integer
Dim WithinLocalNextRangeInput As Integer
Dim LinkNextOutput  As Integer
Dim LinkNextInput  As Integer
Dim ForceOutput As Integer
Dim DontForceOutput As Integer

'Variables for long term memory
Dim LTMemory(Enumber, Enumber) As Integer

'Victor addition to Coco 20 atelier 7-6-2008
'Variables for phase separation
Dim StuckInLoop As Integer
Dim StuckInDistalLoop As Integer
Dim StuckInLocalLoop As Integer
Dim NewModulusConnection As Integer
Dim ModulusRandomElement As Integer
Dim ModuloReceiver As Integer
```

```
Dim ModuloDonor As Integer
Dim ModuloGroup As Integer
'END Victor addition to Coco 20 atelier 7-6-2008

'Victor addition to Coco 20 atelier 28-6-2008
'This is to test for double entry in the Activity Register
Dim DoubleEntry As Integer
'END Victor addition to Coco 20 atelier 28-6-2008

Dim ZeroNSRTotal As Integer
Dim UnusedTopNow As Integer
Dim UnusedTopNext As Integer
Dim InitialTopNow As Integer
Dim InitialTopNext As Integer

'For rewarding and punishing
Dim RewardInputtoOutput As Integer
Dim PunishInputtoOutput As Integer
Dim RewardOutputtoOutput As Integer
Dim PunishOutputtoOutput As Integer

Dim SumNow As Integer
Dim SumNext As Integer
Dim MeanNow As Integer
Dim MeanNext As Integer
Dim InputNowScore(Enumber * Knumber) As Integer
Dim InputNextScore(Enumber * Knumber) As Integer

Dim OldOutputLine As Integer
Dim GoodNewOutput As Integer
Dim GoodOldOutput As Integer
```

```
'for phase separation of outputs
Dim TenNowSet(Enumber) As Integer
Dim TwentyNowSet(Enumber) As Integer
Dim TenNextSet(Enumber) As Integer
Dim TwentyNextSet(Enumber) As Integer
Dim PreviousLine As Integer
Dim TenMinusTwentyNow As Integer
Dim TenMinusTwentyNext As Integer

'For recording successes
Dim RecordPointer As Integer
Dim SuccessTable(2000, 3)
Dim OutputResult As Integer
Dim PunishNextLine As Integer
Dim ShortTermMemoryLength As Integer
Dim FullSuccessStory As Integer

Dim YinYang As Integer
Dim FailureRecordForYinYang As Integer
Dim NumberOfYinYangs As Integer
Dim YinYangCounter As Integer

Dim ExistingNowLink As Integer
Dim ExistingNextLink As Integer

'Each loop counter is used in ONLY one loop

Dim I0 As Integer
Dim I1 As Integer

Dim I2 As Integer
Dim J2 As Integer
```

```
Dim I3 As Integer

Dim I4 As Integer
Dim J4 As Integer
Dim I5 As Integer
Dim I6 As Integer
Dim I7 As Integer
Dim J7 As Integer
Dim I8 As Integer
Dim J8 As Integer
Dim I9 As Integer
Dim J9 As Integer
Dim I10 As Integer
Dim J10 As Integer
Dim I11 As Integer
Dim J11 As Integer
Dim I12 As Integer
Dim I13 As Integer
Dim I14 As Integer
Dim I15 As Integer
Dim I16 As Integer
Dim I17 As Integer
Dim I18 As Integer
Dim J18 As Integer
Dim I19 As Integer
Dim J19 As Integer
Dim I20 As Integer
Dim J20 As Integer
Dim I21 As Integer
Dim I22 As Integer
Dim I23 As Integer
```

```
Dim J23 As Integer
Dim I24 As Integer
Dim J24 As Integer
Dim I25 As Integer
Dim J25 As Integer
Dim I26 As Integer
Dim J26 As Integer

Dim I27 As Integer
Dim I28 As Integer
Dim I29 As Integer
Dim I30 As Integer
Dim I31 As Integer
Dim I32 As Integer
Dim I33 As Integer
Dim I34 As Integer
Dim J34 As Integer
Dim I35 As Integer
Dim J35 As Integer
Dim I36 As Integer
Dim J36 As Integer
Dim I37 As Integer
Dim I38 As Integer
Dim I39 As Integer
Dim I40 As Integer
Dim J40 As Integer
Dim I41 As Integer
Dim I42 As Integer
Dim J42 As Integer
Dim I43 As Integer
Dim J43 As Integer
Dim I44 As Integer
```

```
Dim J44 As Integer
Dim I45 As Integer
Dim J45 As Integer
Dim I46 As Integer
Dim J46 As Integer
Dim K46 As Integer
Dim I47 As Integer
Dim J47 As Integer
Dim I48 As Integer
Dim J48 As Integer
Dim I49 As Integer
Dim J49 As Integer
Dim I50 As Integer
Dim J50 As Integer
Dim I51 As Integer
Dim J51 As Integer
Dim I52 As Integer
Dim J52 As Integer
Dim I53 As Integer
Dim I54 As Integer
Dim J54 As Integer
Dim K54 As Integer
Dim I55 As Integer
Dim I56 As Integer
Dim I57 As Integer
Dim I58 As Integer
Dim I59 As Integer
Dim J59 As Integer
Dim I60 As Integer
Dim J60 As Integer
Dim I61 As Integer
Dim I62 As Integer
```

```
Dim I63 As Integer
Dim J63 As Integer
Dim K63 As Integer
Dim L63 As Integer

Dim I64 As Integer
Dim J64 As Integer
Dim I65 As Integer
Dim J65 As Integer
Dim K65 As Integer
Dim I66 As Integer
Dim J66 As Integer
Dim K66 As Integer
Dim I67 As Integer
Dim J67 As Integer
Dim I68 As Integer
Dim J68 As Integer
Dim I69 As Integer
Dim I70 As Integer
Dim J70 As Integer
Dim I71 As Integer
Dim K71 As Integer
Dim I72 As Integer
Dim K72 As Integer
Dim I73 As Integer
Dim I74 As Integer
Dim I75 As Integer
Dim I76 As Integer
Dim J76 As Integer
Dim I77 As Integer
Dim J77 As Integer
Dim I78 As Integer
```

```
Dim J78 As Integer
Dim I79 As Integer
Dim J79 As Integer
Dim I80 As Integer
Dim I81 As Integer
Dim I82 As Integer
Dim I83 As Integer
Dim I84 As Integer
Dim J84 As Integer
Dim I85 As Integer
Dim I86 As Integer
Dim J86 As Integer
Dim K86 As Integer
Dim I87 As Integer
Dim J87 As Integer
Dim I88 As Integer
Dim J88 As Integer
Dim I89 As Integer
Dim J89 As Integer
Dim I90 As Integer
Dim J90 As Integer
Dim I91 As Integer
Dim J91 As Integer
Dim I92 As Integer
Dim J92 As Integer
Dim K92 As Integer
Dim I93 As Integer
Dim I94 As Integer
Dim J94 As Integer
Dim K94 As Integer
Dim I95 As Integer
Dim J95 As Integer
```

```
Dim I96 As Integer
Dim J96 As Integer
Dim I97 As Integer
Dim I98 As Integer
Dim J98 As Integer
Dim I99 As Integer

'R is for random
Dim R As Integer
Dim R0 As Integer
Dim R1 As Integer
Dim R2 As Integer
Dim R3 As Integer
Dim R4 As Integer
Dim R5 As Integer
Dim R6 As Integer
Dim R7 As Integer
Dim R8 As Integer
Dim R9 As Integer
Dim R10 As Integer
Dim R11 As Integer
Dim R12 As Integer
Dim R13 As Integer
Dim R14 As Integer
Dim R15 As Integer
Dim R16 As Integer
Dim R17 As Integer
Dim R18 As Integer
Dim R19 As Integer
Dim R20 As Integer
Dim R21 As Integer
Dim R22 As Integer
```

```
Dim R23 As Integer
Dim R24 As Integer
Dim R25 As Integer
Dim R26 As Integer
Dim R27 As Integer
Dim R28 As Integer
Dim R29 As Integer
Dim R30 As Integer
Dim R31 As Integer
Dim R32 As Integer
Dim R33 As Integer
Dim R34 As Integer
Dim R35 As Integer
Dim R36 As Integer
'Victor addition to Coco 20 atelier 12-6-2008
Dim R37 As Integer
Dim R38 As Integer
Dim R39 As Integer
Dim R40 As Integer
Dim R41 As Integer
Dim R42 As Integer
Dim R43 As Integer
Dim R44 As Integer
Dim R45 As Integer
Dim R46 As Integer
Dim R47 As Integer
Dim R48 As Integer
Dim R49 As Integer
Dim R50 As Integer
Dim R51 As Integer
Dim R52 As Integer
Dim R53 As Integer
```

```
Dim R54 As Integer
Dim R55 As Integer
Dim R56 As Integer
Dim R57 As Integer
Dim R58 As Integer
Dim RandomElement As Integer
Dim R60 As Integer
Dim R61 As Integer
Dim R62 As Integer
Dim R63 As Integer
Dim R64 As Integer
Dim R65 As Integer
Dim R66 As Integer
Dim R67 As Integer
Dim R68 As Integer
Dim R69 As Integer
Dim R70 As Integer
Dim R71 As Integer

'End Victor addition to Coco 20 atelier 12-6-2008

Dim OnlyScoreOneNow As Integer
Dim OnlyScoreOneNext As Integer
Dim AfterInputPosition As Integer
Dim AvailablePosition As Integer
Dim Sofar As Integer
Dim Sofartemp As Integer
Dim RewardNow As Integer
Dim RewardNext As Integer

Dim NumberOfOutputs As Integer
Dim RecordSuccess As Long
```

```
Dim RecordFailure As Long
Dim InputNeeded As Integer
Dim OutputLinePlusOne As Integer
Dim InputLine As Integer
Dim Reward As Integer
Dim GrowthResponse As Integer
Dim SporulationResponse As Integer
Dim SubtractionOfLines As Integer
Dim StartLoop As Integer
Dim EndLoop As Integer
Dim ActRegLine As Integer
Dim TooBiased As Integer
Dim ForcedOutput As Integer
'

' %%%%%%%%%%%%%%%%%%%%%%%%%%%%%%%%%%%%%%%%% Maurice - 24-
05-2008 commande Pause
%%%%%%%%%%%%%%%%%%%%%%%%%%%%%%%%%%%%%%%%%%%%%%%%
Private Sub Cmd_Susp_Click()                                                    '%
   'Susp = 1 - Susp                                                         '%
End Sub                                                                    '%
'
%%%%%%%%%%%%%%%%%%%%%%%%%%%%%%%%%%%%%%%%%%%%%%%%%
%%%%%%%%%%%%%%%%%%%%%%%%%%%%%%%%%%%%%%%%%%%%%%%%%
%%%%%%%%%%%%%%%%%%%%%%%%%%%%%

Private Sub Enumber_txt_Change()
If Enumber.Text = "" Then
    Enumber.Text = 1
    Exit Sub
End If
If (Val(Enumber) < 1) Then Enumber.Text = 1
If (Val(Enumber) > 30000) Then Enumber.Text = 30000
```

```
End Sub




' %%%%%%%%%%%%%%%%%%%%%%%%%%%%%%%%%%%%%%% Maurice - 29-05-2008 commande Stop
%%%%%%%%%%%%%%%%%%%%%%%%%%%%%%%%%%%%%%%%%%%%%%%%%

Private Sub Stop_Coco_Click()                                          '%

   Stopper = 1 - Stopper                                            '%

End Sub                                                           '%

'
%%%%%%%%%%%%%%%%%%%%%%%%%%%%%%%%%%%%%%%%%%%%%%%%%
%%%%%%%%%%%%%%%%%%%%%%%%%%%%%%%%%%%%%%%%%%%%%%%%%
%%%%%%%%%%%%%%%%%%%%%%%%%%%%%


Private Sub Bigloop0Max_Change()

If Bigloop0Max.Text = "" Then

    Bigloop0Max.Text = 1

    Exit Sub

End If

If (Val(Bigloop0Max) < 1) Then Bigloop0Max.Text = 1

If (Val(Bigloop0Max) > 32755) Then Bigloop0Max.Text = 32755

End Sub

'



Private Sub Command1_Click()          ' Command1 = Go     %%%% Maurice 03-06-2008 - Vrai début du déroulement du code

'

'
%%%%%%%%%%%%%%%%%%%%%%%%%%%%%%%%%%%%%%%%%%%%%%%%%
%%%%%%%%%%%%%%%%%%%%%%%%%%%%%%%%%%%%%%%%%%%%%%%%%
%%%%%%%%%%%%%%%%%%%%%%%%%%%%%%

'

'Show          ' %%%% Maurice 03-06-2008 - inutile, car 'Visible=On' est positionné dans les propriétés de la feuille          '%
```

```
'
Initialize
' %%%%%%%%%%%%%%%%%%%%%%%%%%%% Maurice - 29-05-2008 - Initialisation à partir
de l'écran %%%%%%%%%%%%%%%%%%%%%%%%%%%%%%%%%%%%%%%%%%%%%%%%%%
'   Enumber = Enumber_txt.Text         ' Impossible tant que ces variables sont définies
'%
'   Knumber = Knumber_txt.Text         ' en contantes, ce qui est obligatoire car elles
'%
'   Anumber = Anumber_txt.Text         ' apparaissent ddans des Dim(variable).
'%
'   DowntimeNumber = DownTime_txt.Text ' Reste à étudier un autre système !
'%
'
%%%%%%%%%%%%%%%%%%%%%%%%%%%%%%%%%%%%%%%%%%%%%%%%%%%%%%%%%%%%%%%%%%%%%%%%%%%%%%%%%%%%%%%%%%%%%%%%%%%%%%%%%%%%%%%%%%%%%%%%%%%%%%%%%%%%
For I0 = 1 To MetaLoop
Compute
'ShowResults
'Reflect - this will include condensation and expansion
Next I0
   Maurice_AffTbÉlémFinProg           ' %%%% Maurice - 04-06-2008 - vers affichage en fin de run
du tableau des éléments
'
End Sub


Public Sub ShowResults()
                                                  ' Repère 10
   I12 = 0
   For I12 = 1 To InputRange
'      AInput(I12 - 1) = InputA(I12)
   Next I12
   Label4.Caption = ConstantInput
'
   Maurice_AffTbActivity              ' %%%% Maurice - 04-06-2008 - vers affichage en fin de run du
tableau Activity
```

```
'
   I16 = 0
   For I16 = 1 To Anumber
      If I16 > 13 Then GoTo MissNextLabel 'This is the current number of textbooks
      LHighestNext1(I16 - 1) = SavedHighestNext(I16, 1)
      LHighestNext2(I16 - 1) = SavedHighestNext(I16, 2)
MissNextLabel:
   Next I16
'
I16 = 0
   For I16 = 1 To Anumber
      If I16 > 13 Then GoTo MissNowLabel
      LHighestNow1(I16 - 1) = SavedHighestNow(I16 + 1, 1)
      LHighestNow2(I16 - 1) = SavedHighestNow(I16 + 1, 2)
MissNowLabel:
   Next I16

   LRecordSuccess = RecordSuccess
   LRecordFailure = RecordFailure

   ConnectivityExtractSub
   ConnectivityBinSub
   ConnectivityDisplaySub

'
End Sub

Public Sub ConnectivityExtractSub()
'This gives the number of times each element is cited in the Now fields and in the Next fields of all the Enumber elements
'Signing is ignored!

For I98 = 1 To Enumber
```

```
ConnectivityNow(I98) = 0
ConnectivityNext(I98) = 0
Next I98

'this scoring does not take account of signs
For I98 = 1 To Enumber
    For J98 = 1 To Knumber
    ConnectivityNow(Element(I98, J98)) = ConnectivityNow(Element(I98, J98)) + 1
    Next J98

    For J98 = (Knumber + 1) To (2 * Knumber)
    ConnectivityNext(Element(I98, J98)) = ConnectivityNext(Element(I98, J98)) + 1
    Next J98
Next I98

End Sub

Public Sub ConnectivityBinSub()
'There are connectivity scores for each element.
'This finds how many times a particular score occurs
'and can bin them if the denominator is set to more than 1

ZeroNow = 0
ZeroNext = 0

I98 = 0
J98 = 0

For I98 = 1 To (Knumber * Anumber) 'scores are unlikely to exceed this
BinNow(I98) = 0
BinNext(I98) = 0
Next I98
```

```
    For I98 = 1 To Enumber
    BinnedScore = Int((ConnectivityNow(I98)) / 1)
        Select Case BinnedScore
        Case Is <= 0
        ZeroNow = ZeroNow + 1
        Case Else
        BinNow(BinnedScore) = BinNow(BinnedScore) + 1
        End Select

        BinnedScore = Int((ConnectivityNext(I98)) / 1)
        Select Case BinnedScore
        Case Is <= 0
        ZeroNext = ZeroNext + 1
        Case Else
        BinNext(BinnedScore) = BinNext(BinnedScore) + 1
        End Select



    Next I98

End Sub

Public Sub ConnectivityDisplayInitializeSub()

    Picture3.Scale (-5, (Enumber / 10))-((100 + 20), -20) '(left x, top y) - (right x, bottom y)
    Picture3.Cls
    Picture3.FillStyle = 0

    Picture4.Scale (-5, (Enumber / 10))-((100 + 20), -20) '(left x, top y) - (right x, bottom y)
    Picture4.Cls
    Picture4.FillStyle = 0
```

```
Picture5.Scale (-2, (30))-((100 + 20), -2) '(left x, top y) - (right x, bottom y)
Picture5.Cls
Picture5.FillStyle = 0

Picture6.Scale (-2, (30))-((100 + 20), -2) '(left x, top y) - (right x, bottom y)
Picture6.Cls
Picture6.FillStyle = 0

Picture3.Line (0, 1)-(100, 1) 'x-axis normal
Picture3.Line (0, Enumber / 10)-(0, 0) 'y-axis
Picture4.Line (0, 1)-(100, 1) 'x-axis
Picture4.Line (0, Enumber / 10)-(0, 0) 'y-axis
Picture5.Line (-1, 0)-(90, 0) 'x-axis 'log
Picture5.Line (-1, 200)-(-1, 0) 'y-axis
Picture6.Line (-1, 0)-(90, 0) 'x-axis 'log
Picture6.Line (-1, 200)-(-1, 0) 'y-axis

For I1 = 1 To (Enumber / 2) Step Enumber / 100
Picture3.Line (-2, I1)-(0, I1) 'y-axis marks
Picture4.Line (-2, I1)-(0, I1) 'y-axis marks
Next I1

For I1 = 1 To 100 Step 10
Picture3.Line (I1, 0)-(I1, -3) 'x-axis marks
Picture4.Line (I1, 0)-(I1, -3) 'x-axis marks
Next I1


'Picture3.Circle (0, 0), 1
'Picture4.Circle (0, 0), 1
```

```
    For I1 = 1 To 100 'At present BinNow() is a bin of 1

        Select Case BinNow(I1) 'The radius of the circle is small if the bin is empty
        Case Is = 0
        'Picture3.Circle (I1, BinNow(I1)), 1 / 100 'displays bins e.g., 4 lots of sizes (as determined by ActualMassBinSize)
        'Picture5.Circle (I1, BinNow(I1)), 1 / 100
        Case Is > 0
        Picture3.Circle (I1, BinNow(I1)), 1 / 2 'displays bins e.g., 4 lots of sizes (as determined by ActualMassBinSize)
        Picture5.Circle (I1, Log(BinNow(I1))), 1 / 2
        End Select

    Next I1

    For I1 = 1 To 100

        Select Case BinNext(I1) 'The radius of the circle is small if the bin is empty
        Case Is = 0
        'Picture4.Circle (I1, BinNext(I1)), 1 / 100 'displays bins e.g., 4 lots of sizes (as determined by ActualMassBinSize)

        Case Is > 0
        Picture4.Circle (I1, BinNext(I1)), 1 / 2 'displays bins e.g., 4 lots of sizes (as determined by ActualMassBinSize)
        Picture6.Circle (I1, Log(BinNext(I1))), 1 / 2
        End Select

    Next I1
    DoEvents
End Sub

Public Sub ConnectivityDisplaySub()
```

```
Picture3.Scale (-5, (Enumber / 10))-((100 + 20), -20) '(left x, top y) - (right x, bottom y)
Picture3.Cls
Picture3.FillStyle = 0

Picture4.Scale (-5, (Enumber / 10))-((100 + 20), -20) '(left x, top y) - (right x, bottom y)
Picture4.Cls
Picture4.FillStyle = 0

Picture5.Scale (-2, (30))-((100 + 20), -2) '(left x, top y) - (right x, bottom y)
Picture5.Cls
Picture5.FillStyle = 0

Picture6.Scale (-2, (30))-((100 + 20), -2) '(left x, top y) - (right x, bottom y)
Picture6.Cls
Picture6.FillStyle = 0

Picture3.Line (0, 1)-(100, 1) 'x-axis normal
Picture3.Line (0, Enumber / 10)-(0, 0) 'y-axis
Picture4.Line (0, 1)-(100, 1) 'x-axis
Picture4.Line (0, Enumber / 10)-(0, 0) 'y-axis
Picture5.Line (-1, 0)-(90, 0) 'x-axis 'log
Picture5.Line (-1, 200)-(-1, 0) 'y-axis
Picture6.Line (-1, 0)-(90, 0) 'x-axis 'log
Picture6.Line (-1, 200)-(-1, 0) 'y-axis

For I1 = 1 To (Enumber / 2) Step Enumber / 100
Picture3.Line (-2, I1)-(0, I1) 'y-axis marks
Picture4.Line (-2, I1)-(0, I1) 'y-axis marks
Next I1

For I1 = 1 To 100 Step 10
Picture3.Line (I1, 0)-(I1, -3) 'x-axis marks
```

```
    Picture4.Line (I1, 0)-(I1, -3) 'x-axis marks
    Next I1

    For I1 = 1 To 100

        Select Case BinNow(I1) 'The radius of the circle is small if the bin is empty
        Case Is = 0
        Picture3.Circle (I1, BinNow(I1)), 1 / 100 'displays bins e.g., 4 lots of sizes (as determined by ActualMassBinSize)
        'Picture5.Circle (I1, BinNow(I1)), 1 / 100
        Case Is > 0
        Picture3.Circle (I1, BinNow(I1)), 1 / 2 'displays bins e.g., 4 lots of sizes (as determined by ActualMassBinSize)
        Picture5.Circle (I1, Log(BinNow(I1))), 1 / 2
        End Select

    Next I1

    For I1 = 1 To 100

        Select Case BinNext(I1) 'The radius of the circle is small if the bin is empty
        Case Is = 0
        Picture4.Circle (I1, BinNext(I1)), 1 / 100 'displays bins e.g., 4 lots of sizes (as determined by ActualMassBinSize)

        Case Is > 0
        Picture4.Circle (I1, BinNext(I1)), 1 / 2 'displays bins e.g., 4 lots of sizes (as determined by ActualMassBinSize)
        Picture6.Circle (I1, Log(BinNext(I1))), 1 / 2
        End Select

    Next I1
    DoEvents
End Sub
```

```
Public Sub Initialize()

    Randomize
          ' Repère 30
    RunWithoutInput = 0

    CompatibilitySub 'TestCompatibilitySub3 ' 'TestCompatibilitySub2 '
    RecordSuccess = 0
    RecordFailure = 0
    InputNeeded = 1

'Randomly fills fields of elements with addresses of other elements
'note that arrays are array(row, column)ie Element(Enumber,Knumber)
    I18 = 1
    J18 = 1
    For I18 = 1 To Enumber
        For J18 = 1 To Knumber
InitializeAgain:

            R51 = Int((Enumber * Rnd) + 1)

            Select Case I18 'avoid connecting 1,2 or 3 to outputs

            Case Is <= InputRange
            If R51 > (Enumber - OutputRange) Then GoTo InitializeAgain



            Case Is > (Enumber - OutputRange)
            If R51 <= InputRange Then GoTo InitializeAgain

            Case Else
```

```
            End Select

            Element(I18, J18) = R51

        Next J18
    Next I18

    For I18 = 1 To Enumber
        For J18 = Knumber To (2 * Knumber)
InitializeAgain2:

            R51 = Int((Enumber * Rnd) + 1)

            Select Case I18 'avoid connecting 1,2 or 3 to outputs

            Case Is <= InputRange
            If R51 > (Enumber - OutputRange) Then GoTo InitializeAgain2

            Case Is > (Enumber - OutputRange)
            If R51 <= InputRange Then GoTo InitializeAgain2

            Case Else

            End Select

            Element(I18, J18) = R51

        Next J18
    Next I18
```

```
   'Adds outputs in an initial seeding
   'For I18 = 0 To (OutputRange - 1)
   'For J18 = 1 To 10
    '   Randomize
     '     R63 = Int(((Enumber - OutputRange) * Rnd)) + 1
      '    Element(R63, Knumber + 1) = Enumber - I18
   'Next J18
   'Next I18



'Randomly gives signs to connections at positive:negative ratio of 9:1
For I40 = 1 To Enumber
 For J40 = 1 To Enumber
 SignElement(I40, J40) = 1
           'Randomize
           R15 = Int((100 * Rnd) + 1)
           If R15 > 10 Then SignElement(I40, J40) = 1 Else SignElement(I40, J40) = -1
 Next J40

Next I40

40                                                   ' Repère 40



   'RemoveSelfingSub

'Removes inputs that occur in the
'initialisation step and that might be confusing
   'RemoveSpuriousInputSub
```

```
   I21 = 0
'Load Activity Register with zeroes
   For linenumber = 1 To EndOfActivityRegister
      For I21 = 1 To Anumber
         Activity(linenumber, I21) = 0
      Next
   Next

   linenumber = 1
   NewLineNumber = 2

'set first line of Activity register to random values (but not inputs!)
50                                              ' Repère 50

'TEST, OVERWRITE THIS in testsetups

ReLoadFirstLine:
   I22 = 0
   For I22 = 1 To Anumber
SetUpCycle:
      'Randomize
      Activity(linenumber, I22) = Int((Enumber * Rnd) + 1)
      If Activity(linenumber, I22) <= InputRange Then GoTo SetUpCycle
   Next

I10 = 0
   DoubleEntry = 0
    For I10 = 1 To Anumber - 1
      For J10 = I10 + 1 To Anumber
      If Activity(linenumber, I10) = Activity(linenumber, J10) Then DoubleEntry = DoubleEntry + 1
      If DoubleEntry = 0 Then GoTo Noproblem
```

```
        DoubleEntry = DoubleEntry + 10
Noproblem:
        Next J10
        Next I10

If DoubleEntry > 0 Then GoTo ReLoadFirstLine

'Gives initial CycleElement
CycleElement = InputRange + 1

'gives initial inputs

I10 = 0
    For I10 = 1 To InputRange
    InputA(I10) = 0
    Next I10

    InputA(1) = 1
    InputTotal = 1
    ConstantInput = 1

'This is for testing with defined connections
    'If Test = 1 Then OldTestSetUp
   ' TestEnhanceSub ' remove

    CounterForPreviousSuccess = 1
RunningScoreWindow = 10 'REMOVE AND RESTORE =10
RecordPointer = 1
SuccessTable(RecordPointer, 1) = 1 'inputline
SuccessTable(RecordPointer, 2) = 1 'outputline
SuccessTable(RecordPointer, 3) = 0
```

```
ConnectivityExtractSub
ConnectivityBinSub
ConnectivityDisplayInitializeSub

End Sub

Public Sub TestEnhanceSub()

I79 = 0
J79 = 0

For I79 = 20 To (Enumber - 10) Step 10

Element(I79 + 1, 1) = (I79 + 2)
Element(I79 + 2, 1) = (I79 + 3)
Element(I79 + 3, 1) = (I79 + 1)
Element(I79 + 1, 2) = (I79 + 2)
Element(I79 + 2, 2) = (I79 + 3)
Element(I79 + 3, 2) = (I79 + 1)
Element(I79 + 1, 3) = (I79 + 2)
Element(I79 + 2, 3) = (I79 + 3)
Element(I79 + 3, 3) = (I79 + 1)
Next I79


End Sub

Public Sub YinYangSub()
'YinYang with odds and evens

If NumberOfYinYangs > 1 Then GoTo MissYinYang
Select Case YinYang
```

```
Case Is = 6
For I63 = 4 To Enumber Step 2
For J63 = 4 To Enumber Step 2
CompatibilityTable(I63, J63) = 2 '
Next J63
Next I63

Case Is = 7
For I63 = 5 To Enumber Step 2
For J63 = 5 To Enumber Step 2
CompatibilityTable(I63, J63) = 2 '
Next J63
Next I63

End Select

'makes full incompatibilities between inputs
For I63 = 1 To 3
For J63 = (I63 + 1) To 3
CompatibilityTable(I63, J63) = 0 ' was 1 remove
CompatibilityTable(J63, I63) = 0 ' was 1 remove
Next J63
Next I63

'makes full compatibilities between inputs and elements
For I63 = 1 To Enumber
CompatibilityTable(I63, 1) = 1
CompatibilityTable(1, I63) = 1
CompatibilityTable(I63, 2) = 1
CompatibilityTable(2, I63) = 1
CompatibilityTable(I63, 3) = 1
```

```
CompatibilityTable(3, I63) = 1
Next I63

'makes full incompatibilities between inputs
For I63 = 1 To 3
For J63 = (I63 + 1) To 3
CompatibilityTable(I63, J63) = 0 ' was 1 remove
CompatibilityTable(J63, I63) = 0 ' was 1 remove
Next J63
Next I63

'makes full compatibilities between outputs and elements
For I63 = 1 To Enumber
For J63 = 1 To OutputRange
CompatibilityTable(I63, (1 + Enumber - OutputRange)) = 1
CompatibilityTable((1 + Enumber - OutputRange), I63) = 1
Next J63
Next I63

'Diagnonal selfing compatibilities
For I63 = 1 To Enumber
CompatibilityTable(I63, I63) = 1 'was 10
Next I63

MissYinYang:

End Sub

Public Sub ResetYinYangSub()
For I63 = 1 To Enumber
For J63 = 1 To Enumber
CompatibilityTable(I63, J63) = 1
```

```
Next J63
Next I63

'makes full incompatibilities between inputs
For I63 = 1 To 3
For J63 = (I63 + 1) To 3
CompatibilityTable(I63, J63) = 0 ' was 1 remove
CompatibilityTable(J63, I63) = 0 ' was 1 remove
Next J63
Next I63
End Sub
Public Sub CompatibilitySub()
'At the moment only the inputs are incompatible with one another

'originally made random compatibility groups between 1 and 10 for all elements except for inputs
'now makes everything compatible with everything
For I63 = 1 To Enumber
For J63 = (I63 + 1) To (Enumber - 1)
'randomize
CompatibilityTable(I63, J63) = 1 'was Int((10 * Rnd) + 1)
CompatibilityTable(J63, I63) = 1 ' was = CompatibilityTable(I63, J63)
Next J63
Next I63

'makes full compatibilities for inputs and all other elements upto first half of Enumber
For I63 = 1 To InputRange
For J63 = (InputRange + 1) To Enumber / 2
CompatibilityTable(I63, J63) = 1  'was 10
CompatibilityTable(J63, I63) = 1 'was 10
Next J63
Next I63
```

```
'could make incompatibilies for inputs with second half of elements but doesn't
For I63 = 1 To InputRange
For J63 = ((Enumber / 2) + 1) To Enumber
CompatibilityTable(I63, J63) = 1
CompatibilityTable(J63, I63) = 1
Next J63
Next I63

'makes full incompatibilities between inputs
For I63 = 1 To InputRange
For J63 = (I63 + 1) To InputRange
CompatibilityTable(I63, J63) = 0 ' was 1 remove
CompatibilityTable(J63, I63) = 0 ' was 1 remove
Next J63
Next I63

'Diagnonal selfing compatibilities
For I63 = 1 To Enumber
CompatibilityTable(I63, I63) = 1 'was 10
Next I63

End Sub

Public Sub TestSetUpWinningSeries2()

End Sub


Public Sub WhatShouldWeDoSub()
'Temp1 = HighestNext(1, 1)
'Temp2 = HighestNext(2, 1)
'Temp3 = HighestNext(3, 1)
```

```
'Temp4 = HighestNext(4, 1)
'Temp5 = HighestNext(5, 1)
'Temp6 = HighestNext(6, 1)
'Temp7 = HighestNext(7, 1)
'Temp8 = HighestNext(8, 1)
'Temp9 = HighestNext(9, 1)
'Temp10 = HighestNext(10, 1)
'Temp11 = HighestNext(1, 2)
'Temp12 = HighestNext(2, 2)
'Temp13 = HighestNext(3, 2)
'Temp14 = HighestNext(4, 2)
'Temp15 = HighestNext(5, 2)
'Temp16 = HighestNext(6, 2)
'Temp17 = HighestNext(7, 2)
'Temp18 = HighestNext(8, 2)
'Temp19 = HighestNext(9, 2)
'Temp20 = HighestNext(10, 2)

End Sub



Public Sub Compute() '4/02/2011



'this Bigloop comprises most of the program; it starts by loading inputs


'    Dim Fin_affiche As String                  04-06-2008
    Picture1.Line (0, 0)-(0, 0)
    EnumberLabel.Text = Enumber
    KnumberLabel.Text = Knumber
    AnumberLabel.Text = Anumber
    DownTimeLabel = DowntimeNumber
```

```
For Bigloop0 = 1 To Bigloop0Max '0

If Bigloop0 > 15000 Then RunWithoutInput = 1

LBoucle = Bigloop0
'ShowResults

Maurice_AffTbÉlémPause      ' Maurice 03-06-2008 - Paquet1 des Maurice en fin de programme

'RemoveSelfingSub 'eliminate self-referencing

'RemoveSpuriousInputSub'eliminates spurious input. if I leave this in, the program cannot learn

'DynamicConnectionsSub

'MutationSub

'If Bigloop0 Mod 4 = 1 Then CountOutputLinksSub
'Select Case Bigloop0

'Case Is < 300

'Case Else

'If GoodNewOutput = 0 Then FailureRecordForYinYang = FailureRecordForYinYang + 1
'If FailureRecordForYinYang = 10 Then YinYangSub
'If FailureRecordForYinYang = 10 Then FailureRecordForYinYang = 1

'End Select

  'Clear NewLine
```

```
        I31 = 0
        For I31 = 1 To Anumber
        Activity(NewLineNumber, I31) = 0
        Next I31

    If DowntimeNumber > 0 Then InactivateElementsSub

    'CycleLimit = 10 * Int(Enumber / 10)

    If CyclePermission = 1 Then CycleSub

    If RunWithoutInput = 1 Then GoTo NoInput

    Select Case InputNeeded

    Case Is = 0

    Case Is = 1

    'randomize
    R66 = R66 + 1 'Int((4 * Rnd) + 1)
    If R66 = 1 Then InputSub
    If R66 = 1 Then R66 = 0
    End Select

NoInput:

    'If InputNeeded = 1 Then InputSub 'along with InputAverageConnectivitySub puts
InputNextScore(1 0r 2 0r 3) to a high value

    NextExtractionSub 'puts Next scores from elements in Activity(Linenumber) into the
NextScoreRegister
```

```
    'EmergenceSub is not used here even if there is an input or a cyclic element and the first HighestNext has yet to be loaded

    'and Activity(NewLine, 1) = 0.


    'The following is because EmergentNextScoreRegister has not been filled in EmergenceSub

      I4 = 0

       For I4 = 1 To Enumber

       EmergentNextScoreRegister(I4) = NextScoreRegister(I4) 'EmergentNextScoreRegister is used by the following NextOrderSub

       Next I4


    NextOrderSub

    'ScrambleHighestNextSub

    'WhatShouldWeDoSub

    FindFirstAvailablePositionSub 'FINDS FIRST AVAILABLE POSITION IN ACTIVITY REGISTER, loads HighestNext as Sofar


SavedHighestNext(1, 1) = HighestNext(1, 1)

SavedHighestNext(1, 2) = HighestNext(1, 2)

'SavedHighestNow(1, 1) = HighestNow(1, 1) If left in, this gives the last Now of the previous line in AR

'SavedHighestNow(1, 2) = HighestNow(1, 2)

70                                              ' Repère 70


'testsection

'For I4 = 1 To Enumber

'For J4 = 1 To Knumber * 2

'If Element(I4, J4) = 0 Then WhatShouldWeDoSub

'Next J4

'Next I4



SofarSub 'LOAD ACTIVITY REGISTER USING NOW/NEXT COMPETITION BY OBTAINING HIGHEST NOW EACH CYCLE
```

```
'CHECK THAT THERE ARE NO ZEROES IN ACTIVITY REGISTER ELSE WILL GET AN ERROR FROM element (A1, A2) SINCE A2 WILL EQUAL ZERO'

120                                   ' Repère 120

If OutputNeeded = 1 Then ForcedOutputSub 'remove?

'RemoveZeroAndDoubleEntrySub

'eliminates a second input from Coco
If RemoveInputByCoco = 1 Then RemoveInputGenerationByCocoSub

'sets variables to zero
  Reward = 0
  RewardNow = 0
  RewardNext = 0

DetectionYinYangSub
YinYangSub

If InputNeeded = 0 Then DetectionOutputSub 'detects the presence, nature and number of outputs

'ShowResults 'disable this and enable it in SofarSub if you want to follow loading the ActivityRegister step by step

'LongTermMemorySub

UpdateLineNumberSub
140                                   ' Repère 140
'This is to avoid dividing by zeroes
  If RecordSuccess = 0 Then RecordSuccess = 1
  If RecordFailure = 0 Then RecordFailure = 1
```

```
   Picture1.Line -((Bigloop0 / Bigloop0Max) * 100, RecordSuccess / (RecordSuccess +
RecordFailure) * 100), QBColor(0)
145
   LRecordSuccess = RecordSuccess
   LRecordFailure = RecordFailure

   DoEvents
     'Bigloop0 = 2000
     If Bigloop0 = 1 Then ShowResults
     If Bigloop0 < 3 Then Maurice_AffTbÉlémFinProg

     If Bigloop0 < (Bigloop0Max - 16) Then GoTo DontShowYet



      ShowResults
      If Bigloop0 = (Bigloop0Max - 1) Then Maurice_AffTbÉlémFinProg
      If Bigloop0 = (Bigloop0Max - 1) Then WhatShouldWeDoSub

DontShowYet:

Next Bigloop0    'end of bigloop0         ' Maurice 05-06-2008 - Pour s'assurer que le Next est bien
attribué à Bigloop0 - Repère Paquet

WhatShouldWeDoSub
ShowResults

End Sub

Public Sub RemoveZeroAndDoubleEntrySub()
I10 = 0
   For I10 = 1 To Anumber
      'randomize
```

```
        If Activity(NewLineNumber, I10) = 0 Then Activity(NewLineNumber, I10) = Int((Enumber * Rnd) + 1)
    Next I10

RerunDoubleEntry:

    I10 = 0
    DoubleEntry = 0
     For I10 = 1 To Anumber - 1
        For J10 = I10 + 1 To Anumber
        If Activity(NewLineNumber, I10) = Activity(NewLineNumber, J10) Then DoubleEntry = DoubleEntry + 1
        If DoubleEntry = 0 Then GoTo Noproblem
        Activity(NewLineNumber, I10) = Int((Enumber * Rnd) + 1)

        DoubleEntry = DoubleEntry + 10

Noproblem:
        Next J10
        Next I10

        If DoubleEntry > 0 Then GoTo RerunDoubleEntry
        'If DoubleEntry > 0 Then WhatShouldWeDoSub
End Sub
Public Sub FindFirstAvailablePositionSub()

'after this routine, Sofar points to a filled position
Sofar = 0
    I5 = 0
    For I5 = 1 To Anumber
        AvailablePosition = 0
        If Activity(NewLineNumber, I5) = 0 Then AvailablePosition = 1
        'By inactivating the following line, I take control away from the previous AR Line
```

```
        If AvailablePosition = 1 Then Activity(NewLineNumber, I5) = HighestNext(1, 1) 'There should ALWAYS be a HighestNext to insert

        If AvailablePosition = 1 Then Sofar = I5
        If AvailablePosition = 1 Then I5 = Anumber
    Next I5

End Sub
Public Sub SofarSub()

'Sofar points to a filled space in the Activity Register
    For Sofartemp = Sofar To (Anumber - 1)

Select Case Activity(NewLineNumber, Sofar)

Case Is <= InputRange 'The element is an input

 I62 = 0
    For I62 = 1 To InputRange
    InputNowScore(I62) = 0
    InputNextScore(I62) = 0
    Next I62

Case Else

End Select

NowExtractionSub
'NextExtractionSub 'I am unsure why this is here because this has been done in Compute

'If OutputNeeded = 1 Then ForcedOutputSub 'remove and restore this, maybe ...
```

```
R50 = Int((20 * Rnd) + 1)
If NoiseLevel < R50 Then GoTo MakeNoNoise

'If (RunningScoreTotal / RunningScoreWindow) > (1 / 4) Then GoTo MakeNoNoise

    'randomize
    R50 = Int(RunningScoreWindow * Rnd) - 1 'make this - 1 if want to avoid all Noise after successful learning

    If (RunningScoreTotal / RunningScoreWindow) > (R50 / RunningScoreWindow) Then GoTo MakeNoNoise

NoiseSub

MakeNoNoise:

EmergenceSub

DoubleEntrySub

NowOrderSub
'WhatShouldWeDoSub
'ScrambleHighestNowSub 'This is to avoid the artefact in which Nows with the same score always have the lowest address on top

NextOrderSub
'WhatShouldWeDoSub
'ScrambleHighestNextSub

SavedHighestNext(Sofartemp + 1, 1) = HighestNext(1, 1)
```

```
SavedHighestNext(Sofartemp + 1, 2) = HighestNext(1, 2)
SavedHighestNow(Sofartemp + 1, 1) = HighestNow(1, 1)
SavedHighestNow(Sofartemp + 1, 2) = HighestNow(1, 2)


'ShowResults 'enable this and disable it in SofarSub if you want to follow loading the ActivityRegister step by step


110                                        ' Repère 110

   If HighestNext(1, 2) > (NowNextWeighting * HighestNow(1, 2)) Then Activity(NewLineNumber, Sofar + 1) = HighestNext(1, 1) Else Activity(NewLineNumber, Sofar + 1) = HighestNow(1, 1)

TempSuccessfulConnectionsSub

   Sofar = Sofar + 1

'End of Sofartemp loop
Next Sofartemp


End Sub

Public Sub DoubleEntrySub()
I9 = 0
J9 = 0

   For I9 = 1 To Enumber
      For J9 = 1 To Sofar
         If I9 = Activity(NewLineNumber, J9) Then NowScoreRegister(I9) = 0
         If I9 = Activity(NewLineNumber, J9) Then NextScoreRegister(I9) = 0
         If I9 = Activity(NewLineNumber, J9) Then EmergentNowScoreRegister(I9) = 0
```

```
            If I9 = Activity(NewLineNumber, J9) Then EmergentNextScoreRegister(I9) = 0

        Next J9


    Next I9

End Sub


Public Sub TemporaryResetNowScoreRegisterSub() 'Check
this!!!!!!!!!!!!!!!!!!!!!!!!!!!!!!!!!!!!!!!!!!!!!!!!!!!!!!!!!!!!11




For J9 = 1 To Sofar

            If Activity(NewLineNumber, J9) = 0 Then WhatShouldWeDoSub

         Next J9


'improve this

For I67 = 1 To Sofar


J67 = Activity(NewLineNumber, I67)

NowScoreRegister(J67) = 0


Next I67


End Sub


Public Sub TemporaryResetNextScoreRegisterSub()

For I67 = 1 To Sofar


J67 = Activity(NewLineNumber, I67)

NextScoreRegister(J67) = 0


Next I67


NextOrderSub
```

```
End Sub

Public Sub DetectionYinYangSub()

NumberOfYinYangs = 0
YinYang = 0
   I11 = 0
   For I11 = 1 To Anumber
      If Activity(NewLineNumber, I11) = 6 Then
            NumberOfYinYangs = NumberOfYinYangs + 1
            YinYang = 26
      Else
            If Activity(NewLineNumber, I11) = 7 Then
                        NumberOfYinYangs = NumberOfYinYangs + 1
                        YinYang = 27
            End If
      End If

   Next I11

Select Case NumberOfYinYangs

Case Is = 0

YinYangCounter = YinYangCounter - 1

Case Is = 1

YinYangCounter = 5

Case Else
```

```
YinYangCounter = YinYangCounter - 1

End Select

If YinYangCounter < 0 Then YinYangCounter = 0

Select Case YinYangCounter

Case Is = 0
'do nothing

Case Is = 1
'reset
ResetYinYangSub

Case Is = 5
YinYangSub

End Select

End Sub

Public Sub CountOutputLinksSub()

For I92 = 1 To Enumber
OutputFrequency(I92) = 0
Next I92

'randomize
R69 = Int(Enumber * Rnd) + 1
```

```
For I92 = R69 To Enumber

For J92 = 1 To 2 * Knumber

For K92 = 1 To OutputRange

'randomize

R67 = Int(Enumber * Rnd) + 1

If Element(I92, J92) <> Enumber + 1 - K92 Then GoTo KeepCounting

If Element(I92, J92) = Enumber + 1 - K92 Then OutputFrequency(Enumber + 1 - K92) =
OutputFrequency(Enumber + 1 - K92) + 1

'If OutputFrequency(Enumber + 1 - K92) > OutputRangeMax And SpareLink(I92, J92) = 0 Then
Element(I92, J92) = R67

KeepCounting:

Next K92

Next J92

Next I92


For I92 = 1 To R69

For J92 = 1 To 2 * Knumber

For K92 = 1 To OutputRange

'randomize

R67 = Int(Enumber * Rnd) + 1

If Element(I92, J92) <> Enumber + 1 - K92 Then GoTo KeepOnCounting

If Element(I92, J92) = Enumber + 1 - K92 Then OutputFrequency(Enumber + 1 - K92) =
OutputFrequency(Enumber + 1 - K92) + 1

'If OutputFrequency(Enumber + 1 - K92) > OutputRangeMax And SpareLink(I92, J92) = 0 Then
Element(I92, J92) = R67

KeepOnCounting:

Next K92

Next J92

Next I92



'For I93 = 1 To OutputRange

'Do

'Randomize
```

```
'R67 = Int(Enumber * Rnd) + 1

'R68 = Int(Knumber * Rnd) + 1

'If OutputFrequency(Enumber + 1 - I93) < OutputRangeMin Then Element(R67, R68) = Enumber + 1 - I93

'If OutputFrequency(Enumber + 1 - I93) < OutputRangeMin Then OutputFrequency(Enumber + 1 - I93) = OutputFrequency(Enumber + 1 - I93) + 1

'Loop Until OutputFrequency(Enumber + 1 - I93) >= OutputRangeMin

'Next I93


End Sub



Public Sub ExemptSuccessfulOutputLinksSub()


For I94 = 1 To Enumber

For J94 = 1 To Enumber

SpareLink(I94, J94) = SpareLink(I94, J94) - 1

If SpareLink(I94, J94) < 0 Then SpareLink(I94, J94) = 0

Next J94

Next I94



For I94 = 1 To OutputRange

For J94 = 1 To Anumber

If OutputA(I94) = 1 And Activity(NewLineNumber, J94) = (1 + Enumber) - I94 Then LinkTo = Activity(NewLineNumber, J94)

Next J94

Next I94


For J94 = 1 To Anumber

LinkFrom = Activity(NewLineNumber, J94)

For K94 = 1 To Knumber

If Element(LinkFrom, K94) = LinkTo Then SpareLink(LinkFrom, LinkTo) = 2
```

```
Next K94
Next J94

For J94 = 1 To Anumber
LinkFrom = Activity(NewLineNumber - 1, J94)
For K94 = 1 To Knumber
If Element(LinkFrom, Knumber + K94) = LinkTo Then SpareLink(LinkFrom, LinkTo) = 2
Next K94
Next J94

End Sub

Public Sub DetectionOutputSub()
   NumberOfOutputs = 0




I11 = 0
   For I11 = 1 To Anumber
   For J11 = 1 To OutputRange
       If Activity(NewLineNumber, I11) = (1 + Enumber) - J11 Then NumberOfOutputs =
NumberOfOutputs + 1
       Next J11
   Next I11

I11 = 0
   For I11 = 1 To Anumber
       A = Activity(NewLineNumber, I11)
       UnusedElement(A, 2) = UnusedElement(A, 2) + 20
       UsedElement(A, 2) = UsedElement(A, 2) + 20
   Next I11
```

```
    OutputLinePlusOne = NewLineNumber + 1
    If OutputLinePlusOne = EndOfActivityRegister + 1 Then OutputLinePlusOne = 1
'InputLine has been set by the InputSubroutine
130                                         ' Repère 130

    Select Case NumberOfOutputs

    Case 0
    'There is no output (NumberOfOutputs = 0) and there are 2 possibilities:

        Select Case OutputLinePlusOne

        Case InputLine
            '1/ The Activity Register is full and OutputLinePlusOne = InputLine
            InputNeeded = 1
            RecordFailure = RecordFailure + 1

        Case Else
            '2/ The Activity Register is not full and OutputLinePlusOne <> InputLine

            InputNeeded = 0
                'randomize
    OutputLacking = OutputLacking + 1
    'OutputFrequency = OutputFrequency + 1
    'If OutputFrequency = 3 Then OutputFrequency = 0
    If OutputLacking > ForcedOutputProbability Then OutputNeeded = 1 '


'If ForcedOutputProbability > OutputFrequency Then InputNeeded = 1

        End Select
```

```
    Case 1
    'There is a single output - but is it the desired one?
      'SuccessTableBasedDecisionRewardOrPunishSub 'remove
      TestDecisionRewardOrPunishSub 'remove
      InputNeeded = 1
       OutputLacking = 0

    Case Else
    'There is more than one output so there must be punishment
       'SuccessTableBasedDecisionRewardOrPunishSub 'remove
       TestDecisionRewardOrPunishSub 'remove
       'PhaseSeparationSub
       InputNeeded = 1
       OutputLacking = 0 'remove

       DoEvents
    End Select
End Sub




Public Sub ForcedOutputSub()

DontForceOutput = 0 'new routine to remove

For I78 = 1 To Anumber
For J78 = 1 To OutputRange
If Activity(NewLineNumber, I78) = (1 + Enumber - J78) Then DontForceOutput = 1
Next J78
Next I78

If DontForceOutput = 1 Then GoTo MissForcingOutput
```

```
'remove the above
'randomize
R54 = Int(OutputRange * Rnd)
Activity(NewLineNumber, Anumber) = Enumber - R54 'CHECK THIS!!!!!!!!!!

MissForcingOutput:

OutputNeeded = 0

End Sub
Public Sub NextExtractionSub()

'Extracts how often an address has been referred to in the Next field of the elements active in the Activity Register.
'linenumber selects 'the line in the Activity Register and I2 selects the element within it; J+Knumber selects the elements within its Next field

'I1 Sets NextScoreRegister and HighestNext to zero
I1 = 0
  For I1 = 1 To Enumber
    NextScoreRegister(I1) = 0
    HighestNext(I1, 1) = 0
    HighestNext(I1, 2) = 0
  Next I1

I2 = 0
J2 = 0
S = 0
For I2 = 1 To Anumber
  A = Activity(linenumber, I2)

 'No scoring from outputs
```

```
        If A > Enumber - OutputRange Then GoTo NoScoringOfOutputNexts1

'No scoring from inputs of 1, 2 and 3
        'If A = 1 Then GoTo NoScoringOfOutputNexts1
        'If A = 2 Then GoTo NoScoringOfOutputNexts1
         'If A = 3 Then GoTo NoScoringOfOutputNexts1

  For J2 = 1 To Knumber
     'randomize
     If Element(A, J2 + Knumber) = 0 Then Element(A, J2 + Knumber) = Int((Enumber * Rnd) + 1)

'No scoring from Next fields containing inputs of 1, 2 and 3
        'If Element(A, J2 + Knumber) = 1 Then GoTo NoScoringOfOutputNexts2
        'If Element(A, J2 + Knumber) = 2 Then GoTo NoScoringOfOutputNexts2
         'If Element(A, J2 + Knumber) = 3 Then GoTo NoScoringOfOutputNexts2

If Downtime(Element(A, J2 + Knumber)) > 0 Then GoTo NoScoringOfOutputNexts2

'Stop cyclic element being loaded twice
If Element(A, J2 + Knumber) = CycleElement Then GoTo NoScoringOfOutputNexts2
S = Element(A, J2 + Knumber)

Sign = SignElement(A, Element(A, J2 + Knumber))
If Sign >= 0 Then NextScoreRegister(S) = NextScoreRegister(S) + 1
If Sign < 0 Then NextScoreRegister(S) = NextScoreRegister(S) - 1

NoScoringOfOutputNexts2:
```

```
    Next J2

NoScoringOfOutputNexts1:

Next I2

I61 = 0
For I61 = 1 To InputRange
If InputA(I61) = 1 Then NextScoreRegister(I61) = NextScoreRegister(I61) + InputNextScore(I61)
Next I61

'Ensure that a Next that is ALREADY in the ActivityRegister does not get scored (and perhaps loaded again)
I77 = 0
    For I77 = 1 To Sofar
    A = Activity(NewLineNumber, I77)
    If A = 0 Then GoTo DontResetNextScore
    NextScoreRegister(A) = 0
DontResetNextScore:
    Next I77

'ReverseDownTimeSub 'REMOVEREVERSENEXTS

End Sub

Public Sub NowExtractionSub()
'FIND HIGHEST NOW VALUES FROM ELEMENTS IN ACTIVITY AND LOAD INTO NowScoreRegister

'Sets NowScoreRegister and Highest Now to zero
```

```
        I6 = 0
        For I6 = 1 To Enumber
            NowScoreRegister(I6) = 0
            HighestNow(I6, 1) = 0
            HighestNow(I6, 2) = 0
        Next I6

'(2)Extracts how often an address has been referred to in the Now field
'of the elements active (7 when full) in the Activity Register

I7 = 0
J7 = 0
For I7 = 1 To Sofar
    A = Activity(NewLineNumber, I7)
     'Do not score Output fields
        If A > Enumber - OutputRange Then GoTo NoScoringOfOutputNows

        'Do not count the Nows of inputs
        'If A = 1 Then GoTo NoScoringOfInputNows
        'If A = 2 Then GoTo NoScoringOfInputNows
        'If A = 3 Then GoTo NoScoringOfInputNows

        'randomize
        If A = 0 Then A = Int((Enumber * Rnd) + 1)
        For J7 = 1 To Knumber
        If Element(A, J7) = 0 Then Element(A, J7) = Int((Enumber * Rnd) + 1)

        If Downtime(Element(A, J7)) > 0 Then GoTo NoScoringOfNows


        S = Element(A, J7)
        Sign = SignElement(A, S)
```

```
If Sign > 0 Then NowScoreRegister(S) = NowScoreRegister(S) + 1
If Sign < 0 Then NowScoreRegister(S) = NowScoreRegister(S) - 1


NoScoringOfNows:

   Next J7
NoScoringOfOutputNows:
NoScoringOfInputNows:

Next I7

I61 = 0
For I61 = 1 To InputRange
If InputA(I61) = 1 Then NowScoreRegister(I61) = NowScoreRegister(I61) + InputNowScore(I61)
Next I61

'Ensure that a Now that is ALREADY in the ActivityRegister does not get scored (and perhaps loaded again)
   I76 = 0
   For I76 = 1 To Sofar
    A = Activity(NewLineNumber, I76)
    If A = 0 Then WhatShouldWeDoSub
   If A = 0 Then GoTo DontResetNowScore
   NowScoreRegister(A) = 0
DontResetNowScore:
   Next I76

ReverseDownTimeSub

End Sub

Public Sub ReverseDownTimeSub()
```

```
'the idea is to avoid compressing the thoughts
'by preventing the elements in line t+1 from being loaded into the previous line t
'this entails taking the elements being loaded into the NewLine and ensuring that the addresses in their Nexts,
'which may correspond to elements often present in the following line, are not loaded
  I76 = 0
  J76 = 0
   For I76 = 1 To Sofar
    A = Activity(NewLineNumber, I76)
   For J76 = 1 To Knumber
   'If A = 0 Then GoTo NoReverseDowntime 'REMOVEREVERSENEXTS
   NowScoreRegister(Element(A, Knumber + J76)) = 0
   'NextScoreRegister(Element(A, Knumber + J76)) = 0 'REMOVEREVERSENEXTS
   Next J76
   Next I76
'NoReverseDowntime: 'REMOVEREVERSENEXTS
End Sub
Public Sub NextOrderSub()

'(3)use NextScoreRegister so most frequent are ordered in HighestNext

80                                          ' Repère 80

   TempHighestNext(1) = 0
   TempHighestNext(2) = 0

   I4 = 0
   For I4 = 1 To Enumber
   HighestNext(I4, 1) = I4
   HighestNext(I4, 2) = EmergentNextScoreRegister(I4) 'These are the scores modified by EmergenceSub
   Next I4
```

```
I4 = 0
J4 = 0
For I4 = 1 To (Enumber - 1)
For J4 = (I4 + 1) To Enumber
If HighestNext(I4, 2) >= HighestNext(J4, 2) Then GoTo KeepHighestNext
TempHighestNext(1) = HighestNext(I4, 1)
TempHighestNext(2) = HighestNext(I4, 2)
HighestNext(I4, 1) = HighestNext(J4, 1)
HighestNext(I4, 2) = HighestNext(J4, 2)
HighestNext(J4, 1) = TempHighestNext(1)
HighestNext(J4, 2) = TempHighestNext(2)

KeepHighestNext:
Next J4
Next I4
'WhatShouldWeDoSub

End Sub

Public Sub EmergenceSub()

For I64 = 1 To Sofar
For J64 = 1 To Enumber
If Activity(NewLineNumber, I64) = 0 Then GoTo ZeroAddressError 'this is an error condition

Select Case CompatibilityTable(Activity(NewLineNumber, I64), J64)

Case Is < 1
EmergentNowScoreRegister(J64) = NowScoreRegister(J64) * 0 'remove l
EmergentNextScoreRegister(J64) = NextScoreRegister(J64) * 1 'remove 1

Case Is = 1
```

```
EmergentNowScoreRegister(J64) = NowScoreRegister(J64) * 1 'was * 1 / 2
EmergentNextScoreRegister(J64) = NextScoreRegister(J64) * 1 'was * 1 / 2
Case Is = 2
EmergentNowScoreRegister(J64) = NowScoreRegister(J64) * 10 'was * 1 / 2
EmergentNextScoreRegister(J64) = NextScoreRegister(J64) * 10 'was * 1  / 2
Case Is = 3
EmergentNowScoreRegister(J64) = NowScoreRegister(J64) * 1 'was * 1 / 2
EmergentNextScoreRegister(J64) = NextScoreRegister(J64) * 1 'was * 1  / 2
Case Is = 4
EmergentNowScoreRegister(J64) = NowScoreRegister(J64) * 1 'was * 1 / 2
EmergentNextScoreRegister(J64) = NextScoreRegister(J64) * 1 'was * 1 / 2
Case Is = 5
EmergentNowScoreRegister(J64) = NowScoreRegister(J64) * 1 'was * 1  / 2
EmergentNextScoreRegister(J64) = NextScoreRegister(J64) * 1 'was * 1 / 2


Case Is = 6
EmergentNowScoreRegister(J64) = NowScoreRegister(J64) * 1 'was 2
EmergentNextScoreRegister(J64) = NextScoreRegister(J64) * 1 'was 2
Case Is = 7
EmergentNowScoreRegister(J64) = NowScoreRegister(J64) * 1 'was 2
EmergentNextScoreRegister(J64) = NextScoreRegister(J64) * 1 'was 2
Case Is = 8
EmergentNowScoreRegister(J64) = NowScoreRegister(J64) * 1 'was 2
EmergentNextScoreRegister(J64) = NextScoreRegister(J64) * 1 'was 2
Case Is = 9
EmergentNowScoreRegister(J64) = NowScoreRegister(J64) * 1 'was 2
EmergentNextScoreRegister(J64) = NextScoreRegister(J64) * 1 'was 2

Case Is > 9
EmergentNowScoreRegister(J64) = NowScoreRegister(J64) * 1 'was 3
EmergentNextScoreRegister(J64) = NextScoreRegister(J64) * 1 'was 3
```

```
'If CompatibilityTable(Activity(NewLineNumber, I64), J64) < 8 Then NowScoreRegister(J64) = 0 'try NSR = NSR*table

'If CompatibilityTable(Activity(NewLineNumber, I64), J64) < 8 Then NextScoreRegister(J64) = 0


End Select

Next J64

Next I64


ZeroAddressError:


End Sub


Public Sub NowOrderSub() '7/02/2011


TempHighestNow(1) = 0

TempHighestNow(2) = 0


        I8 = 0


    For I8 = 1 To Enumber

    HighestNow(I8, 1) = I8

    HighestNow(I8, 2) = EmergentNowScoreRegister(I8)

    Next I8


        I8 = 0

        J8 = 0

For I8 = 1 To (Enumber - 1)

For J8 = (I8 + 1) To Enumber

If HighestNow(I8, 2) >= HighestNow(J8, 2) Then GoTo KeepHighestNow

TempHighestNow(1) = HighestNow(I8, 1)

TempHighestNow(2) = HighestNow(I8, 2)

HighestNow(I8, 1) = HighestNow(J8, 1)
```

```
HighestNow(I8, 2) = HighestNow(J8, 2)
HighestNow(J8, 1) = TempHighestNow(1)
HighestNow(J8, 2) = TempHighestNow(2)

KeepHighestNow:
Next J8

Next I8

End Sub

Public Sub NoiseSub()

'this forcibly inserts a randomly chosen element (that is not an input) into the Activity Register
'R49 = 100 + Int((100) * Rnd) 'to get elements between 100 and Enumber
R49 = Int((Enumber - (InputRange + 1)) * Rnd) + (InputRange + 1) 'to get elements between 1 and Enumber
If R49 > Enumber - OutputRange Then GoTo NoNoise

NowScoreRegister(R49) = 10 * Knumber * Anumber

NoNoise:
End Sub

Public Sub CycleSub()

Cycling = 0
'CycleLength says how many lines of the ActivityRegister there are per insertion of a CycleElement
```

```
CycleStep = CycleStep + 1

If CycleStep < CycleLength Then GoTo MissCycle

If CycleStep = CycleLength Then CycleStep = 0

CycleElement = CycleElement + 1

If CycleElement > Enumber Then CycleElement = InputRange + 1

If CycleElement < (InputRange + 1) Then CycleElement = InputRange + 1

If CycleElement > Enumber - OutputRange Then CycleElement = InputRange + 1

Activity(NewLineNumber, 1) = CycleElement

'Let other routines know a CycleElement has been inserted

Cycling = 1

MissCycle:

End Sub

Public Sub ScrambleHighestNowSub()

'The problem to be solved (if it is a problem) is to prevent the same Nows from being loaded again and again even though

'there are other Nows with the same score. This is because the ordering routine puts the lowest addresses on top. So scramble them.

ScrambleNowNumber = 0

MarkScrambleNowStart = 0

For I49 = 1 To Enumber - 1
```

```
If MarkScrambleNowStart = 0 Then MarkScrambleNowStart = I49

If HighestNow(I49, 2) = HighestNow(I49 + 1, 2) Then ScrambleNowNumber =
ScrambleNowNumber + 1


Select Case ScrambleNowNumber


Case Is = 0

'There is just one line so don't try to scramble!

MarkScrambleNowStart = 0

GoTo ContinueUpdatingNow


Case Is > 0

If HighestNow(I49, 2) = HighestNow(I49 + 1, 2) Then GoTo ContinueUpdatingNow

'Scramble HighestNows with the same score by swapping

    'randomize

    R20 = Int((ScrambleNowNumber * Rnd)) ' check this gives 0 to some number!!!

TempScrambleNowAddress = HighestNow(MarkScrambleNowStart, 1)

TempScrambleNowScore = HighestNow(MarkScrambleNowStart, 2)

HighestNow(MarkScrambleNowStart, 1) = HighestNow(MarkScrambleNowStart + R20, 1)

HighestNow(MarkScrambleNowStart, 2) = HighestNow(MarkScrambleNowStart + R20, 2)

HighestNow(MarkScrambleNowStart + R20, 1) = TempScrambleNowAddress

HighestNow(MarkScrambleNowStart + R20, 2) = TempScrambleNowScore

ScrambleNowNumber = 0

MarkScrambleNowStart = 0


End Select


ContinueUpdatingNow:


Next I49


End Sub
```

```
Public Sub ScrambleHighestNextSub()

'The problem to be solved (if it is a problem) is to prevent the same Nexts from being loaded again and again even though

'there are other Nexts with the same score. This is because the ordering routine puts the lowest addresses on top. So scramble them.

ScrambleNextNumber = 0

MarkScrambleNextStart = 0

TempScrambleNextAddress = Enumber


For I52 = 1 To Enumber - 1

If MarkScrambleNextStart = 0 Then MarkScrambleNextStart = I52

If HighestNext(I52, 2) = HighestNext(I52 + 1, 2) Then ScrambleNextNumber = ScrambleNextNumber + 1


Select Case ScrambleNextNumber


Case Is = 0

'There is just one line so don't try to scramble!

MarkScrambleNextStart = 0

GoTo ContinueUpdatingNext


Case Is > 0

If HighestNext(I52, 2) = HighestNext(I52 + 1, 2) Then GoTo ContinueUpdatingNext

'Scramble HighestNows with the same score by swapping

    'randomize

    R21 = Int((ScrambleNextNumber * Rnd)) ' check this gives 0 to some number!!!

TempScrambleNextAddress = HighestNext(MarkScrambleNextStart, 1)

TempScrambleNextScore = HighestNext(MarkScrambleNextStart, 2)

HighestNext(MarkScrambleNextStart, 1) = HighestNext(MarkScrambleNextStart + R21, 1)

HighestNext(MarkScrambleNextStart, 2) = HighestNext(MarkScrambleNextStart + R21, 2)

HighestNext(MarkScrambleNextStart + R21, 1) = TempScrambleNextAddress

HighestNext(MarkScrambleNextStart + R21, 2) = TempScrambleNextScore

ScrambleNextNumber = 0
```

```
MarkScrambleNextStart = 0

End Select

ContinueUpdatingNext:

Next I52




End Sub

Public Sub InactivateElementsSub()
'This prevents elements that have been active in the AR from being active again
'for a down time

'This allows neurones to recover progressively
    For I38 = 1 To Enumber
        Downtime(I38) = Downtime(I38) - 1
        If Downtime(I38) < 0 Then Downtime(I38) = 0
    Next I38

    For I39 = 1 To Anumber
        A = Activity(linenumber, I39)
        Downtime(A) = DowntimeNumber
    Next I39

'Must not prevent a response else cannot have 10 in successive rows when needed!

For I85 = 1 To OutputRange
Downtime(1 + Enumber - I85) = 0
```

```
Next I85
'What happens if we include inputs?
'Downtime(1) = 0 'remove
'Downtime(2) = 0
'Downtime(3) = 0

End Sub

Public Sub TempSuccessfulConnectionsSub()

If linenumber < 2 Then GoTo MissTempSuccessfulConnections

CitedElement = Activity(NewLineNumber, Sofar + 1) 'This is the latest element to be loaded into the
ActivityRegister

Select Case CitedElement

    Case Is = HighestNow(1, 1) 'if the cited element equals this then strengthen the Now link
    I90 = 0
    J90 = 0

    For I90 = 1 To Sofar
    CitingElement = Activity(NewLineNumber, I90)
        For J90 = 1 To Knumber
        If Element(CitingElement, J90) = CitedElement Then TempMatrixNow(CitingElement,
CitedElement) = 1
        Next J90
    Next I90

    Case Is = HighestNext(1, 1) 'if the cited element equals this then strengthen the Next link
    I90 = 0
    J90 = 0
```

```
    For I90 = 1 To Sofar
    CitingElement = Activity(linenumber, I90)
        For J90 = (Knumber + 1) To 2 * Knumber
        If Element(CitingElement, J90) = CitedElement Then TempMatrixNext(CitingElement,
CitedElement) = 1
        Next J90
    Next I90

End Select

MissTempSuccessfulConnections:

End Sub

Public Sub SuccessfulConnectionsSub()

For I91 = 1 To Enumber
For J91 = 1 To Enumber
MatrixNow(I91, J91) = MatrixNow(I91, J91) + TempMatrixNow(I91, J91)
MatrixNext(I91, J91) = MatrixNext(I91, J91) + TempMatrixNext(I91, J91)
Next J91
Next I91

I91 = 0
J91 = 0

For I91 = 1 To Enumber
For J91 = 1 To Enumber

If MatrixNow(I91, J91) > 10 < 20 Then MatrixNow(I91, J91) = 25
If MatrixNow(I91, J91) = 25 Then GoTo EndMatrixNowUpdate
If MatrixNow(I91, J91) > 19 < 30 Then MatrixNow(I91, J91) = 35
If MatrixNow(I91, J91) = 35 Then GoTo EndMatrixNowUpdate
```

```
If MatrixNow(I91, J91) > 29 < 50 Then MatrixNow(I91, J91) = 45

If MatrixNow(I91, J91) = 45 Then GoTo EndMatrixNowUpdate

If MatrixNow(I91, J91) >= 45 Then MatrixNow(I91, J91) = 60


EndMatrixNowUpdate:


If MatrixNext(I91, J91) > 10 < 20 Then MatrixNext(I91, J91) = 25

If MatrixNext(I91, J91) = 25 Then GoTo EndMatrixNextUpdate

If MatrixNext(I91, J91) > 19 < 30 Then MatrixNext(I91, J91) = 35

If MatrixNext(I91, J91) = 35 Then GoTo EndMatrixNextUpdate

If MatrixNext(I91, J91) > 29 < 50 Then MatrixNext(I91, J91) = 45

If MatrixNext(I91, J91) = 45 Then GoTo EndMatrixNextUpdate

If MatrixNext(I91, J91) >= 49 Then MatrixNext(I91, J91) = 60


EndMatrixNextUpdate:

Next J91

Next I91


For I91 = 1 To Enumber

For J91 = 1 To Enumber

TempMatrixNow(I91, J91) = 0

TempMatrixNext(I91, J91) = 0

Next J91

Next I91


End Sub


'Public Sub SpareSuccessfulConnectionsSub()

'come here from having detected success; we are not actually protecting an existing link but the likelihood of one

'Do the Nows

'For I86 = StartLoop To EndLoop

'For J86 = 1 To Anumber
```

```
'FirstElement = Activity(I86, J86)
'For K86 = 1 To Anumber
'SecondElement = Activity(I86, K86)
'If FirstElement = SecondElement Then GoTo DontSpareNow 'avoids selfing
'MatrixNow(FirstElement, SecondElement) = 7
'MatrixNow(SecondElement, FirstElement) = 7
'DontSpareNow:
'Next K86
'Next J86
'Next I86

'Do the Nexts
'For I86 = StartLoop To (EndLoop - 1)
'For J86 = 1 To Anumber
'FirstElement = Activity(I86, J86)
'For K86 = 1 To Anumber
'SecondElement = Activity(I86 + 1, K86)
'If FirstElement = SecondElement Then GoTo DontSpareNext 'avoids selfing
'MatrixNext(FirstElement, SecondElement) = 7
'DontSpareNext:
'Next K86
'Next J86
'Next I86

'End Sub

Public Sub DynamicConnectionsSub()

For I88 = 1 To Enumber
For J88 = 1 To Enumber
If MatrixNow(I88, J88) > 0 Then MatrixNow(I88, J88) = MatrixNow(I88, J88) - 1
If MatrixNext(I88, J88) > 0 Then MatrixNext(I88, J88) = MatrixNext(I88, J88) - 1
```

```
Next J88
Next I88

For I87 = 1 To Enumber
For J87 = 1 To Knumber
'R64 = Int(Knumber * Rnd) + 1
If R64 > 1 Then GoTo SpareNow

RandomElement = Int(Enumber * Rnd) + 1
If MatrixNow(I87, Element(I87, J87)) > 0 Then GoTo SpareNow
Element(I87, J87) = RandomElement
SpareNow:
Next J87
Next I87

For I89 = 1 To Enumber
For J89 = (Knumber + 1) To (Knumber * 2)
'R65 = Int(Knumber * Rnd) + 1
If R65 > 1 Then GoTo SpareNext

RandomElement = Int(Enumber * Rnd) + 1
If MatrixNext(I89, Element(I89, J89)) > 0 Then GoTo SpareNext
Element(I89, J89) = RandomElement
SpareNext:
Next J89
Next I89

End Sub

Public Sub SuccessTableBasedDecisionRewardOrPunishSub() '25/3/2011
' input sequence is 1, 2, 3, 2, 1, 2, 3 … for outputs 10 20 20 10 10 20 20
'Recall, InputTotal = 1, or 2 for growth (needs 10 as output)
```

```
'and InputTotal = 3 for sporulation (needs 20 as output)
'note that having more than one copy of the right output is also punishable

RewardDecision = 0
RunningScoreAction = 0
GoodNewOutput = 0


    Select Case InputTotal


    Case 1 '1 needs 10
        If GrowthResponse = 1 And SporulationResponse = 0 Then GoodNewOutput = 1

    Case 2  '2 here needs 20'
        If SporulationResponse = 1 And GrowthResponse = 0 Then GoodNewOutput = 1

    Case 3  '3 needs 20
        If SporulationResponse = 1 And GrowthResponse = 0 Then GoodNewOutput = 1

    'Case 4  '2 here needs 10 RESTORE THIS WHEN NO LONGER TESTING
        'If GrowthResponse = 1 And SporulationResponse = 0 Then GoodNewOutput = 1

    End Select

    Select Case GoodNewOutput

    Case Is = 0
    RecordPointer = RecordPointer + 1
SuccessTable(RecordPointer, 1) = InputLine
SuccessTable(RecordPointer, 2) = NewLineNumber
SuccessTable(RecordPointer, 3) = 0
```

```
        RecordFailure = RecordFailure + 1

Case Is = 1
RecordPointer = RecordPointer + 1
SuccessTable(RecordPointer, 1) = InputLine
SuccessTable(RecordPointer, 2) = NewLineNumber
SuccessTable(RecordPointer, 3) = 1
        RecordSuccess = RecordSuccess + 1

End Select

SuccessTableWindow = SuccessTableWindow + 1
If SuccessTableWindow = 1 Then SuccessTableSub 'remove
If SuccessTableWindow = 1 Then SuccessTableWindow = 0



   DoEvents
End Sub

Public Sub TestDecisionRewardOrPunishSub()
RewardDecision = 0
RunningScoreAction = 0
GoodNewOutput = 0

For I84 = 1 To OutputRange
For J84 = 1 To Anumber
If OutputA(I84) = 1 And Activity(NewLineNumber, J84) = (1 + Enumber) - I84 Then
GoodNewOutput = 1
Next J84
Next I84
```

```
 If NumberOfOutputs > 1 Then GoodNewOutput = 0

Select Case GoodNewOutput
Case Is = 0 'CHOICE OF PUNISHMENTS
  LineChoice = NewLineNumber - (OldOutputLine + 1)

  StartLoop = (OldOutputLine + 1) + Int(LineChoice * Rnd)
  EndLoop = StartLoop
  'Select Case NewLineNumber - InputLine
  'StartLoop = OldOutputLine + 1
  'EndLoop = NewLineNumber
  For I81 = 1 To RepeatRewardPunish
  PunishMutateNowSub

  StartLoop = EndLoop - 1
  PunishMutateNextSub
  Next I81
  'Case Is >= 3
  'StartLoop = InputLine
  'EndLoop = NewLineNumber - 2 'a new and dangerous change!
  'PunishMutateNowSub
  'StartLoop = InputLine - 1 'remove this addition to Coco64?
  'PunishMutateNextSub
  'EndLoop = NewLineNumber - 1
  'Case Is = 2
  'StartLoop = InputLine
  'EndLoop = NewLineNumber - 1 'a new and dangerous change!
  'PunishMutateNowSub
  'StartLoop = InputLine - 1 'remove this addition to Coco64?
```

```
 'PunishMutateNextSub
 'Case Is = 1
 'StartLoop = InputLine
 'EndLoop = NewLineNumber 'a new and dangerous change!
 'PunishMutateNowSub
 'StartLoop = InputLine - 1 'remove this addition to Coco64?
 'PunishMutateNextSub
 'Case Is = 0
 'StartLoop = InputLine
 'EndLoop = NewLineNumber 'a new and dangerous change!
 'PunishMutateNowSub
 'PunishMutateNowSub
 'StartLoop = InputLine - 1 'remove this addition to Coco64?
 'PunishMutateNextSub
 'PunishMutateNextSub

 'End Select
 RecordFailure = RecordFailure + 1
 'CyclePermission = 1 'REMOVECYCLEPERMISSION

Case Is = 1

SuccessfulConnectionsSub
'ExemptSuccessfulOutputLinksSub

StartLoop = InputLine
EndLoop = NewLineNumber

 For I81 = 1 To RepeatRewardPunish
ShortRewardNowSub
' ExecuteRandomRewardNowSub 'remove
   Next I81
```

```
    If GoodOldOutput = 1 Then StartLoop = OldOutputLine Else StartLoop = InputLine 'remove this addition to Coco64?

        For I81 = 1 To RepeatRewardPunish
        ShortRewardNextSub
        Next I81

        'ExecuteRandomRewardNextSub



        'Protect likely successful links from dynamic overwriting
        'StartLoop = InputLine
        'EndLoop = NewLineNumber

        RecordSuccess = RecordSuccess + 1

        'CyclePermission = 0 'REMOVECYCLEPERMISSION
End Select

 GoodOldOutput = GoodNewOutput
 OldOutputLine = NewLineNumber

'This is for MutationSub
RunningScorePointer = RunningScorePointer + 1
If RunningScorePointer > RunningScoreWindow Then RunningScorePointer = 1
    If GoodNewOutput = 1 Then RunningScore(RunningScorePointer) = 1
    If GoodNewOutput = 0 Then RunningScore(RunningScorePointer) = 0

End Sub
```

```
Public Sub SuccessTableSub()

StartLoop = SuccessTable(RecordPointer - SuccessTableWindow, 1)

For I73 = (RecordPointer - SuccessTableWindow) To RecordPointer
PresentResult = SuccessTable(I73, 3)
SameResult = 0
If SuccessTable(I73, 3) = SuccessTable(I73 + 1, 3) Then SameResult = 1
EndLoop = SuccessTable(I73, 2)

Select Case SameResult

Case Is = 0 'the result is different so reward or punish the previous set

    Select Case PresentResult
    Case Is = 0 'so punish present set
    PunishMutateNowSub
    PunishMutateNextSub
    'give startloop of new set
    'If I73 + 1 = RecordPointer Then GoTo EndSuccessLoop
    StartLoop = SuccessTable(I73 + 1, 1)

    Case Is = 1 'so reward present set
    ShortRewardNowSub
    'ExecuteRandomRewardNowSub ' remove
    ShortRewardNextSub
    'ExecuteRandomRewardNextSub
    'give startloop of new set
    'If I73 + 1 = RecordPointer Then GoTo EndSuccessLoop
    StartLoop = SuccessTable(I73 + 1, 1)
    End Select
```

```
Case Is = 1 'just continue updating unless need to exit
    Select Case RecordPointer
    Case Is = I73
        Select Case PresentResult
        Case Is = 0
        PunishMutateNowSub
        PunishMutateNextSub
        Case Is = 1
        ShortRewardNowSub
        'ExecuteRandomRewardNowSub 'remove
        ShortRewardNextSub
        'ExecuteRandomRewardNextSub
        End Select
    Case Else

    End Select

End Select

EndSuccessLoop:

Next I73

End Sub

Public Sub RecordSuccessSub()

'SuccessTable(RecordPointer, InputPointer, OutputPointer, ResultPointer)
'EarliestSuccessfulInputLine
'LastSuccessfulOutputLine
```

```
ShortTermMemoryLength = 20
FullSuccessStory = 0

StartLoop = SuccessTable(RecordPointer, 1)

I73 = RecordPointer
OutputResult = 1
Do Until OutputResult = 0
StartLoop = SuccessTable(I73, 1)
I73 = I73 - 1
If I73 = RecordPointer - ShortTermMemoryLength Then OutputResult = 0
If I73 = RecordPointer - ShortTermMemoryLength Then FullSuccessStory = 1

OutputResult = SuccessTable(I73, 3)
'PunishNextLine = SuccessTable(I73, 2)
Loop

EndLoop = NewLineNumber

End Sub

Public Sub ShortRewardNowSub()

    If ActivateReward = 0 Then GoTo MissRandomRewardNow

I25 = 0
    J25 = 0

    For ActRegLine = StartLoop To EndLoop

For I25 = 1 To Anumber
```

```
DontOverwriteNow = 0

'randomize
        R6 = Int((Knumber * Rnd) + 1)

AvoidDirectCouplingInputOutput:
AvoidSelfingRandomNow:
        'randomize
        R7 = Int((Anumber * Rnd) + 1)

        FirstElement = Activity(ActRegLine, I25)
        SecondElement = Activity(ActRegLine, R7)

        If FirstElement = SecondElement Then GoTo AvoidSelfingRandomNow
        'ExistingNowLink = 0
        'I35 = 0
        'For I35 = 1 To Knumber
        'If Element(FirstElement, I35) = SecondElement Then ExistingNowLink = 1
        'Next I35
        'If ExistingNowLink = 0 Then GoTo AvoidCouplingInputToSecondHalfEnumberNow:



        TooBiased = 0
        I35 = 0
        For I35 = 1 To Knumber
        If Element(FirstElement, I35) = SecondElement Then TooBiased = TooBiased + 1
        Next I35
        If TooBiased > NowTooBiasedLimit Then GoTo
AvoidCouplingInputToSecondHalfEnumberNow:

        'remove this section
```

```
Select Case FirstElement

Case Is <= InputRange
'If SecondElement = 10 Then GoTo AvoidDirectCouplingInputOutput
'If SecondElement = 20 Then GoTo AvoidDirectCouplingInputOutput
'If SecondElement > Enumber / 2 Then GoTo AvoidCouplingInputToSecondHalfEnumberNow

Case Is > Enumber - OutputRange
' If SecondElement < Enumber / 2 Then GoTo
AvoidCouplingInputToSecondHalfEnumberNow



Case Else

'If FirstElement Mod 2 > SecondElement Mod 2 Then GoTo
AvoidCouplingEvenOddOrOddEven
'If SecondElement Mod 2 > FirstElement Mod 2 Then GoTo
AvoidCouplingEvenOddOrOddEven

End Select

'it needs this to learn properly:
For J25 = 1 To Anumber 'avoid reward routine overwriting a good combination

If Element(FirstElement, R6) = Activity(ActRegLine, J25) Then DontOverwriteNow = 1
'If SignElement(FirstElement, Element(FirstElement, R6)) < 0 Then DontOverwriteNow = 1

Next J25

'randomize

If DontOverwriteNow = 0 Then Element(FirstElement, R6) = SecondElement
```

```
AvoidCouplingInputToSecondHalfEnumberNow:
AvoidCouplingEvenOddOrOddEven:
        Next I25
    Next ActRegLine

MissRandomRewardNow:

End Sub

Public Sub ExecuteRandomRewardNowSub()   '2/02/2011

'reward Now by writing address of one element into the field of another
180                                                    ' Repère 180

    If ActivateReward = 0 Then GoTo MissRandomRewardNow
    'If RunningScoreTotal / RunningScoreWindow > 1 / 2 Then GoTo MissRandomRewardNow
    'If RunningScoreTotal >= RunningScoreWindow Then GoTo MissRandomRewardNow




    I25 = 0
    J25 = 0

    For ActRegLine = StartLoop To EndLoop
For I25 = 1 To Anumber

DontOverwriteNow = 0

'randomize
            R6 = Int((Knumber * Rnd) + 1)

AvoidDirectCouplingInputOutput:
```

```
AvoidSelfingRandomNow:
    'randomize
    R7 = Int((Anumber * Rnd) + 1)

    FirstElement = Activity(ActRegLine, I25)
    SecondElement = Activity(ActRegLine, R7)

    If FirstElement = SecondElement Then GoTo AvoidSelfingRandomNow
    TooBiased = 0
    I35 = 0
    For I35 = 1 To Knumber
    If Element(FirstElement, I35) = SecondElement Then TooBiased = TooBiased + 1
    Next I35
    If TooBiased > NowTooBiasedLimit Then GoTo
AvoidCouplingInputToSecondHalfEnumberNow:

    'remove this section

    Select Case FirstElement

    Case Is = 1
    'If SecondElement = 10 Then GoTo AvoidDirectCouplingInputOutput
    'If SecondElement = 20 Then GoTo AvoidDirectCouplingInputOutput
    'If SecondElement > Enumber / 2 Then GoTo AvoidCouplingInputToSecondHalfEnumberNow

    Case Is = 2
    'If SecondElement = 10 Then GoTo AvoidDirectCouplingInputOutput
    'If SecondElement = 20 Then GoTo AvoidDirectCouplingInputOutput
    'If SecondElement > Enumber / 2 Then GoTo AvoidCouplingInputToSecondHalfEnumberNow

    Case Is = 3
    'If SecondElement = 10 Then GoTo AvoidDirectCouplingInputOutput
```

```
'If SecondElement = 20 Then GoTo AvoidDirectCouplingInputOutput

'If SecondElement > Enumber / 2 Then GoTo AvoidCouplingInputToSecondHalfEnumberNow


Case Is > Enumber - OutputRange

' If SecondElement < Enumber / 2 Then GoTo
AvoidCouplingInputToSecondHalfEnumberNow



Case Else


'If FirstElement Mod 2 > SecondElement Mod 2 Then GoTo
AvoidCouplingEvenOddOrOddEven

'If SecondElement Mod 2 > FirstElement Mod 2 Then GoTo
AvoidCouplingEvenOddOrOddEven


End Select


For J25 = 1 To Anumber 'avoid reward routine overwriting a good combination or a negative
connection


If Element(FirstElement, R6) = Activity(ActRegLine, J25) Then DontOverwriteNow = 1

Next J25


'randomize


If DontOverwriteNow = 0 Then Element(FirstElement, R6) = SecondElement


AvoidCouplingInputToSecondHalfEnumberNow:

AvoidCouplingEvenOddOrOddEven:

Next I25

Next ActRegLine


MissRandomRewardNow:
```

```
End Sub


Public Sub ShortRewardNextSub()

If ActivateReward = 0 Then GoTo MissRandomRewardNext
'If RunningScoreTotal / RunningScoreWindow > 1 / 2 Then GoTo MissRandomRewardNext
'If RunningScoreTotal >= RunningScoreWindow Then GoTo MissRandomRewardNext
    'If DeltaRunningScore > 0 Then GoTo MissRandomRewardNext

    I26 = 0
    J26 = 0
    I35 = 0

    For ActRegLine = StartLoop To (EndLoop - 1)

 For I26 = 1 To Anumber

            DontOverwriteNext = 0

AvoidDirectCouplingInputNextOutput:
            'randomize
            R8 = Int((Knumber * Rnd) + 1)
            'randomize
            R9 = Int((Anumber * Rnd) + 1)

            FirstElement = Activity(ActRegLine, I26)
            SecondElement = Activity(ActRegLine + 1, R9)
            If FirstElement = SecondElement Then GoTo AvoidCouplingNextElementToItself:

           'the following is to test limited but strong Next connections - remove
           'ExistingNextLink = 0
```

```
'I35 = 0
'For I35 = 1 To Knumber
'If Element(FirstElement, I35) = SecondElement Then ExistingNextLink = 1
'Next I35
'If ExistingNextLink = 0 Then GoTo AvoidCouplingNext:

TooBiased = 0
For I35 = 1 To Knumber
  If Element(FirstElement, Knumber + I35) = SecondElement Then TooBiased = TooBiased +
1
Next I35
If TooBiased > NextTooBiasedLimit Then GoTo MakeNoRandomNextReward

          'remove this section

Select Case FirstElement

Case Is <= InputRange
'If SecondElement = 10 Then GoTo AvoidDirectCouplingInputNextOutput
'If SecondElement = 20 Then GoTo AvoidDirectCouplingInputNextOutput

'If SecondElement > Enumber / 2 Then GoTo
AvoidCouplingInputToSecondHalfEnumberNext

Case Is > Enumber - OutputRange
'If SecondElement < Enumber / 2 Then GoTo AvoidCouplingInputToSecondHalfEnumberNext

'Case Else
' If FirstElement Mod 2 = SecondElement Mod 2 Then GoTo
AvoidCouplingEvenEvenOrOddOdd
' If SecondElement Mod 2 = FirstElement Mod 2 Then GoTo
AvoidCouplingEvenEvenOrOddOdd
```

```
End Select

For J26 = 1 To Anumber 'avoid reward routine overwriting a good combination

If Element(FirstElement, R8 + Knumber) = Activity(ActRegLine + 1, J26) Then DontOverwriteNext = 1

'Sign = SignElement(FirstElement, Element(FirstElement, R8 + Knumber))

'If Sign < 0 Then DontOverwriteNext = 1

Next J26

If DontOverwriteNext = 0 Then Element(FirstElement, R8 + Knumber) = SecondElement

AvoidCouplingInputToSecondHalfEnumberNext:

AvoidCouplingNextElementToItself:

AvoidCouplingEvenEvenOrOddOdd:

AvoidCouplingNext:

Next I26

MakeNoRandomNextReward:

Next ActRegLine

MissRandomRewardNext:

End Sub

Public Sub ExecuteRandomRewardNextSub() '2/02/2011

'reward Next

190                              ' Repère 190
```

```
If ActivateReward = 0 Then GoTo MissRandomRewardNext
'If RunningScoreTotal / RunningScoreWindow > 1 / 2 Then GoTo MissRandomRewardNext
'If RunningScoreTotal >= RunningScoreWindow Then GoTo MissRandomRewardNext
    'If DeltaRunningScore > 0 Then GoTo MissRandomRewardNext

    I26 = 0
    J26 = 0
    I35 = 0

    For ActRegLine = StartLoop To (EndLoop - 1)

 For I26 = 1 To Anumber

            DontOverwriteNext = 0

AvoidDirectCouplingInputNextOutput:
            'randomize
            R8 = Int((Knumber * Rnd) + 1)
            'randomize
            R9 = Int((Anumber * Rnd) + 1)

            FirstElement = Activity(ActRegLine, I26)
            SecondElement = Activity(ActRegLine + 1, R9)
            If FirstElement = SecondElement Then GoTo AvoidCouplingNextElementToItself:

           TooBiased = 0
            For I35 = 1 To Knumber
                If Element(FirstElement, Knumber + I35) = SecondElement Then TooBiased = TooBiased +
1
            Next I35
            If TooBiased > NextTooBiasedLimit Then GoTo MakeNoRandomNextReward
```

```
'remove this section

Select Case FirstElement

Case Is = 1
'If SecondElement = 10 Then GoTo AvoidDirectCouplingInputNextOutput
'If SecondElement = 20 Then GoTo AvoidDirectCouplingInputNextOutput

'If SecondElement > Enumber / 2 Then GoTo
AvoidCouplingInputToSecondHalfEnumberNext

Case Is = 2
'If SecondElement = 10 Then GoTo AvoidDirectCouplingInputNextOutput
'If SecondElement = 20 Then GoTo AvoidDirectCouplingInputNextOutput
'If SecondElement > Enumber / 2 Then GoTo AvoidCouplingInputToSecondHalfEnumberNext

Case Is = 3
'If SecondElement = 10 Then GoTo AvoidDirectCouplingInputNextOutput
'If SecondElement = 20 Then GoTo AvoidDirectCouplingInputNextOutput
'If SecondElement > Enumber / 2 Then GoTo AvoidCouplingInputToSecondHalfEnumberNext

Case Is > Enumber - OutputRange
'If SecondElement < Enumber / 2 Then GoTo AvoidCouplingInputToSecondHalfEnumberNext



'Case Else
' If FirstElement Mod 2 = SecondElement Mod 2 Then GoTo
AvoidCouplingEvenEvenOrOddOdd
' If SecondElement Mod 2 = FirstElement Mod 2 Then GoTo
AvoidCouplingEvenEvenOrOddOdd

End Select
```

```
For J26 = 1 To Anumber 'avoid reward routine overwriting a good combination

If Element(FirstElement, R8 + Knumber) = Activity(ActRegLine + 1, J26) Then
DontOverwriteNext = 1

Next J26

If DontOverwriteNext = 0 Then Element(FirstElement, R8 + Knumber) = SecondElement

AvoidCouplingInputToSecondHalfEnumberNext:
AvoidCouplingNextElementToItself:
AvoidCouplingEvenEvenOrOddOdd:

Next I26

MakeNoRandomNextReward:

Next ActRegLine

MissRandomRewardNext:

End Sub
Public Sub ExecuteRandomRewardNextOverlapSub()
'This is needed because when a routine to be rewarded has run off the end of the AR to continue from
its start

200                                     ' Repère 200

If ActivateReward = 0 Then GoTo MissRandomRewardNextOverlap
'If RunningScoreTotal / RunningScoreWindow > 1 / 2 Then GoTo MissRandomRewardNextOverlap
'If RunningScoreTotal >= RunningScoreWindow Then GoTo MissRandomRewardNextOverlap
'If DeltaRunningScore > 0 Then GoTo MissRandomRewardNextOverlap
```

```
I26 = 0
   I35 = 0

 For I26 = 1 To Anumber

           DontOverwriteNextOverlap = 0

           'randomize
           R8 = Int((Knumber * Rnd) + 1)

AvoidDirectCouplingInputOverlapOutput:

           'randomize
           R9 = Int((Anumber * Rnd) + 1)

          TooBiased = 0
           For I35 = 1 To Knumber
               If Element(Activity(StartLoop, I26), Knumber + I35) = Activity(EndLoop, R9) Then
TooBiased = TooBiased + 1
           Next I35
           If TooBiased > NextTooBiasedLimit Then GoTo MakeNoRandomNextOverlapReward

           FirstElement = Activity(StartLoop, I26)
           SecondElement = Activity(EndLoop, R9)
           'Select Case FirstElement

           'Case Is = 1
           'If SecondElement = 10 Then GoTo AvoidDirectCouplingInputOverlapOutput
           'If SecondElement = 20 Then GoTo AvoidDirectCouplingInputOverlapOutput
          'If SecondElement > Enumber / 2 Then GoTo
AvoidCouplingInputToSecondHalfEnumberOverlap
```

```
'Case Is = 2

'If SecondElement = 10 Then GoTo AvoidDirectCouplingInputOverlapOutput

'If SecondElement = 20 Then GoTo AvoidDirectCouplingInputOverlapOutput

'If SecondElement > Enumber / 2 Then GoTo AvoidCouplingInputToSecondHalfEnumberOverlap


'Case Is = 3

'If SecondElement = 10 Then GoTo AvoidDirectCouplingInputOverlapOutput

'If SecondElement = 20 Then GoTo AvoidDirectCouplingInputOverlapOutput

'If SecondElement > Enumber / 2 Then GoTo AvoidCouplingInputToSecondHalfEnumberOverlap


'Case Is = 10

'If SecondElement < Enumber / 2 Then GoTo AvoidCouplingInputToSecondHalfEnumberOverlap


'Case Is = 20

'If SecondElement < Enumber / 2 Then GoTo AvoidCouplingInputToSecondHalfEnumberOverlap


' Case Else


'End Select


For J26 = 1 To Anumber 'avoid reward routine overwriting a good combination


If ActRegLine = EndOfActivityRegister Then GoTo MissEndofRegisterProblem

If Element(Activity(ActRegLine, I26), R8 + Knumber) = Activity(ActRegLine + 1, J26) Then DontOverwriteNextOverlap = 1


MissEndofRegisterProblem:

Next J26
```

```
    If DontOverwriteNextOverlap = 0 Then Element(FirstElement, R8 + Knumber) = 
Activity(EndLoop, R9)

AvoidCouplingInputToSecondHalfEnumberOverlap:

    Next I26

MakeNoRandomNextOverlapReward:
MissRandomRewardNextOverlap:

    End Sub

Public Sub PunishNowLineAllocationSub()
'Since the Activity Register is cyclical, this works out which lines to punish

If ActivatePunish = 0 Then GoTo MissAllPunish 'TESTS EFFECTS OF MISSING PUNISHMENTS

    SubtractionOfLines = NewLineNumber - InputLine
210                                                     ' Repère 210
    Select Case SubtractionOfLines

        'The lines to be punished are in the direction 1 to EndOfActivityRegister e.g. 1000
        Case Is > 0
        StartLoop = InputLine
        EndLoop = NewLineNumber

        PunishMutateNowSub
```

```
        Case Is < 0

        StartLoop = InputLine
        EndLoop = EndOfActivityRegister

        PunishMutateNowSub

        StartLoop = 1
        EndLoop = NewLineNumber

         PunishMutateNowSub


        Case Is = 0

        StartLoop = NewLineNumber
        EndLoop = NewLineNumber

         PunishMutateNowSub


    End Select

MissAllPunish:

    DoEvents
End Sub

Public Sub PunishNextLineAllocationSub()
'Since the Activity Register is cyclical, this works out which lines to punish
```

```
If ActivatePunish = 0 Then GoTo MissAllPunish 'TESTS EFFECTS OF MISSING PUNISHMENTS

    SubtractionOfLines = NewLineNumber - InputLine
210                                                         ' Repère 210
    Select Case SubtractionOfLines

        'The lines to be punished are in the direction 1 to EndOfActivityRegister e.g. 1000
        Case Is > 0
If InputLine - 1 < 1 Then StartLoop = 1
        StartLoop = InputLine - 1
        EndLoop = NewLineNumber

        PunishMutateNextSub

        Case Is < 0
    If InputLine - 1 < 1 Then StartLoop = 1
        StartLoop = InputLine - 1
        EndLoop = EndOfActivityRegister

        PunishMutateNextSub



        StartLoop = EndOfActivityRegister
        EndLoop = 1

        PunishMutateNextOverlap
        'StrongPunishNextOverlap

        StartLoop = 1
        EndLoop = NewLineNumber
```

```
        PunishMutateNextSub



        Case Is = 0

        If InputLine - 1 < 1 Then StartLoop = 1
        StartLoop = InputLine - 1
        EndLoop = NewLineNumber

        PunishMutateNextSub



    End Select

MissAllPunish:

    DoEvents
End Sub





Public Sub DefineInputOutputSub()

'InputRange = 4 is defined in choices
'OutputRange = 2 is defined in choices
'set InputTotalMax to have the size of the pattern
'define inputA(inputrange) as integer
'OutputA(1)= 1 means we want enumber as output
'OutputA(2)= 1 means we want enumber-1 as output
```

```
'OutputA(3)= 1 means we want enumber-2 as output


For I82 = 1 To InputRange
InputA(I82) = 0
Next I82

For I83 = 1 To OutputRange
OutputA(I83) = 0
Next I83

'This gives the input pattern and the corresponding output pattern
Select Case InputTotal '1,2,3,4,5 ...1000,999,998,997,996

Case Is = 1
InputA(1) = 1
OutputA(1) = 1

Case Is = 2
InputA(2) = 1
OutputA(2) = 1

Case Is = 3
InputA(3) = 1
OutputA(2) = 1

Case Is = 4
InputA(2) = 1
OutputA(1) = 1

Case Is = 5
InputA(3) = 1
```

```
OutputA(3) = 1

'Case Is = 6
'InputA(6) = 1
'OutputA(6) = 1

'Case Is = 7
'InputA(7) = 1
'OutputA(7) = 1

'Case Is = 8
'InputA(8) = 1
'OutputA(8) = 1

'Case Is = 9
'InputA(9) = 1
'OutputA(9) = 1
End Select



End Sub

Public Sub InputSub()

'START OF INPUT SECTION
'cross-referencing of inputs has not been eliminated in this program

'constantinput allows input to remain unchanged for x turns
'as far as I can see, it just means skipping the input section
280                                             ' Repère 280
  ConstantInput = ConstantInput + 1
  If ConstantInput < KeepInputSameForThisNumber Then GoTo keepinput
```

```
    If ConstantInput = KeepInputSameForThisNumber Then ConstantInput = 1

'the input pattern is only changed when ConstantInput allows the following

    InputTotal = InputTotal + 1
    If InputTotal = InputTotalMax Then InputTotal = 1

DefineInputOutputSub

keepinput: 'load newline of Activity Register
285                                             ' Repère 285

'If Cycling = 1 there is already an address in the first ‘position of the AR NewLine
    AfterInputPosition = 1
If Cycling = 1 Then AfterInputPosition = 2
    'I32 = 0 ' rather than InputSub itself loading the input, it allows Input to be anticipated via
InputAverageConnectivitySub
    'For I32 = 1 To 3
        'If InputA(I32) = 1 Then
            'Activity(NewLineNumber, AfterInputPosition) = I32
        'If InputA(I32) = 1 Then
            'AfterInputPosition = AfterInputPosition + 1
        'End If
    'Next I32

InputAverageConnectivitySub

InputNeeded = 0
```

```
    InputLine = NewLineNumber


'END OF INPUT SECTION

End Sub





Public Sub InputAverageConnectivitySub()


'The original intention was to allow anticipation by using the Next scores of the previous line - it could still be done

'This gives a score to the input that should allow it to be inserted BUT make sure that the register is not set to zero after this!!!!!!!!!




    I32 = 0

    For I32 = 1 To InputRange

        If InputA(I32) = 1 Then InputNowScore(I32) = (Knumber * Anumber) 'or could be = HighestNow(200, 2)

        If InputA(I32) = 1 Then InputNextScore(I32) = (Knumber * Anumber) * 2 'or could be = HighestNext(20 * Anumber, 2)


    Next I32

End Sub


Public Sub LongTermMemorySub()

'The LTM contains the essence of the Elements table and is a matrix Elements x Elements.

'It is updated depending on RunningScore (i.e. the equivalent of laying down a short term memory) if thngs go well

'It is linked to Downtime so that an important connection is not be disrupted readily

'It can be used to reconstruct the Elements table after a period in which there has been no real progress


'Victor addition 11-06-2008
```

```
For I54 = 1 To Enumber
For J54 = 1 To Enumber
LTMemory(I54, J54) = 0
Next J54
Next I54



For I54 = 1 To Enumber
For K54 = 1 To Knumber
J54 = Element(I54, K54)
'Addition to Coco22 31-07-2008
If J54 = 0 Then GoTo MissZeroElement
LTMemory(I54, J54) = LTMemory(I54, J54) + SignElement(I54, K54)
MissZeroElement:
'End of Addition to Coco22 31-07-2008
Next K54
Next I54

'TestEnhanceSub 'remove
'END of Victor addition 11-06-2008
End Sub

Public Sub UpdateLineNumberSub()
290                                                   ' Repère 290
    linenumber = linenumber + 1
    If linenumber = EndOfActivityRegister + 1 Then linenumber = 1
    NewLineNumber = linenumber + 1
    If linenumber = EndOfActivityRegister Then NewLineNumber = 1
    If linenumber = 1 Then NewLineNumber = 2
End Sub

Public Sub MutationSub()
```

```
    UsedElementSub
    UnusedElementSub

    Select Case Bigloop0

    Case Is < 200

    Case Else

    'There is a common frequency for mutations irrespective of the number of elements
    'so if it is 1/10, we need to allow for different enumbers by calling up the MutationSub
Enumber/100 times

    'This changes the severity of reward and punishment according to recent history
    TimeToUseRunningScore = TimeToUseRunningScore + 1

If TimeToUseRunningScore < RunningScoreWindow Then GoTo TooSoonToMutate
RunningScoreTotal = 0

    For I53 = 1 To RunningScoreWindow
RunningScoreTotal = RunningScoreTotal + RunningScore(I53) 'information comes from
TestDecisionRewardOrPunishSub
Next I53

        Select Case RunningScoreTotal

    Case Is <= RunningScoreWindow - 3
        For I80 = 1 To Enumber / 100
        'randomize
```

```
MutationElement = LowestUsedElement(I80, 1) 'Int((Enumber * Rnd) + 1)
'randomize
MutationPosition = Int((2 * Knumber * Rnd) + 1)
'randomize
MutatedElement = Int((Enumber * Rnd) + 1)
Element(MutatedElement, MutationPosition) = MutationElement
Next I80
'R64 = (Int(Knumber * Rnd) + 1) / 4
'R65 = (Int(Knumber * Rnd) + 1) / 4

'DynamicConnectionsSub
' CountOutputLinksSub

TimeToUseRunningScore = 0

Case Is = RunningScoreWindow - 2
For I80 = 1 To Enumber / 200
'randomize
MutationElement = LowestUsedElement(I80, 1) 'Int((Enumber * Rnd) + 1)
'randomize
MutationPosition = Int((2 * Knumber * Rnd) + 1)
'randomize
MutatedElement = Int((Enumber * Rnd) + 1)
Element(MutatedElement, MutationPosition) = MutationElement
Next I80

'R64 = 2 * Int(Knumber * Rnd) + 1
'R65 = 2 * Int(Knumber * Rnd) + 1

'DynamicConnectionsSub

TimeToUseRunningScore = 0
```

```
Case Is = RunningScoreWindow - 1
For I80 = 1 To Enumber / 400
'randomize
MutationElement = HighestUsedElement(I80, 1) 'Int((Enumber * Rnd) + 1)
A = HighestUsedElement(I80, 2)
'randomize
MutationPosition = Int((2 * Knumber * Rnd) + 1)
'randomize
MutatedElement = Int((Enumber * Rnd) + 1)
Element(MutatedElement, MutationPosition) = MutationElement

'randomize
MutationElement = LowestUsedElement(I80, 1) 'Int((Enumber * Rnd) + 1)
A = LowestUsedElement(I80, 2)
'randomize
MutationPosition = Int((2 * Knumber * Rnd) + 1)
'randomize
MutatedElement = Int((Enumber * Rnd) + 1)
Element(MutatedElement, MutationPosition) = MutationElement
Next I80

'R64 = 10 * Int(Knumber * Rnd) + 1
'R65 = 10 * Int(Knumber * Rnd) + 1

'DynamicConnectionsSub

TimeToUseRunningScore = 0

Case Else
'do nothing RunningScoreTotal=RunningScoreWindow
```

```
        End Select
'runningscoretotal = maximum?
TooSoonToMutate:

End Select

End Sub

Public Sub UsedElementSub()
'Used and Unused elements are scored in the DetectionOutputSub

For I96 = 1 To Enumber 'This is just the labelling so that for element X with a score of Y,
UsedElement(X,1) = X and UsedElement(X,2)=Y
UsedElement(I96, 1) = I96
Next I96

For I96 = 1 To InputRange 'Sets inputs to zero
UsedElement(I96, 2) = 0
Next I96

For I96 = Enumber - (OutputRange + 1) To Enumber 'Sets outputs to zero
UsedElement(I96, 2) = 0
Next I96

For I96 = 1 To Enumber 'reduces the scores of all elements
UsedElement(I96, 2) = UsedElement(I96, 2) - 1
If UsedElement(I96, 2) < 1 Then UsedElement(I96, 2) = 1
Next I96

For I96 = 1 To Enumber 'creates a table
HighestUsedElement(I96, 1) = UsedElement(I96, 1)
HighestUsedElement(I96, 2) = UsedElement(I96, 2)
Next I96
```

```
        I95 = 0
        J95 = 0
For I95 = 1 To Enumber - 1
For J95 = (I95 + 1) To Enumber
If HighestUsedElement(I95, 2) >= HighestUsedElement(J95, 2) Then GoTo KeepHighestUsed
TempUsedElement(1) = HighestUsedElement(I95, 1)
TempUsedElement(2) = HighestUsedElement(I95, 2)
HighestUsedElement(I95, 1) = HighestUsedElement(J95, 1)
HighestUsedElement(I95, 2) = HighestUsedElement(J95, 2)
HighestUsedElement(J95, 1) = TempUsedElement(1)
HighestUsedElement(J95, 2) = TempUsedElement(2)
KeepHighestUsed:
Next J95
Next I95

'scramble - take the first tenth of the elements, choose one at random, and use it to replace
'one of the HighestUsedElements; do this for all of the 1/10 elements
For I95 = 1 To Enumber / 10
'randomize
R71 = Int(Rnd * Enumber / 10) + 1
TempUsedElement(1) = HighestUsedElement(I95, 1)
TempUsedElement(2) = HighestUsedElement(I95, 2)
HighestUsedElement(I95, 1) = HighestUsedElement(R71, 1)
HighestUsedElement(I95, 2) = HighestUsedElement(R71, 2)
HighestUsedElement(R71, 1) = TempUsedElement(1)
HighestUsedElement(R71, 2) = TempUsedElement(2)
If HighestUsedElement(I95, 1) = 0 Then WhatShouldWeDoSub
Next I95

End Sub
Public Sub UnusedElementSub()
```

```
'The UnusedElement matrix has two columns, one corresponding to the address of the element and the other to its score
For I96 = 1 To Enumber
UnusedElement(I96, 1) = I96
Next I96

For I96 = 1 To InputRange
UnusedElement(I96, 2) = Enumber 'This excludes inputs from being considered
Next I96

For I96 = Enumber - (OutputRange + 1) To Enumber
UnusedElement(I96, 2) = Enumber 'This excludes outputs
Next I96

For I96 = 1 To Enumber
UnusedElement(I96, 2) = UnusedElement(I96, 2) - 1
If UnusedElement(I96, 2) < 1 Then UnusedElement(I96, 2) = 1
Next I96

For I96 = 1 To Enumber
LowestUsedElement(I96, 1) = UnusedElement(I96, 1)
LowestUsedElement(I96, 2) = UnusedElement(I96, 2)
Next I96


    I95 = 0
    J95 = 0
For I95 = 1 To Enumber - 1
For J95 = (I95 + 1) To Enumber
If LowestUsedElement(I95, 2) <= LowestUsedElement(J95, 2) Then GoTo KeepLowestUsed
TempUnusedElement(1) = LowestUsedElement(I95, 1)
TempUnusedElement(2) = LowestUsedElement(I95, 2)
```

```
LowestUsedElement(I95, 1) = LowestUsedElement(J95, 1)

LowestUsedElement(I95, 2) = LowestUsedElement(J95, 2)

LowestUsedElement(J95, 1) = TempUnusedElement(1)

LowestUsedElement(J95, 2) = TempUnusedElement(2)

'If LowestUsedElement(I95, 1) = 0 Then WhatShouldWeDoSub

KeepLowestUsed:

Next J95

Next I95


'GoTo MissScramble


'scramble

ScrambleLowestNumber = 0

MarkScrambleLowestStart = 0


For I97 = 1 To Enumber - 1

If MarkScrambleLowestStart = 0 Then MarkScrambleLowestStart = I97

If LowestUsedElement(I97, 2) = LowestUsedElement(I97 + 1, 2) Then ScrambleLowestNumber =
ScrambleLowestNumber + 1


Select Case ScrambleLowestNumber


Case Is = 0

'There is just one line so don't try to scramble!

MarkScrambleLowestStart = 0

GoTo ContinueUpdatingLowest


Case Is > 0

If LowestUsedElement(I97, 2) = LowestUsedElement(I97 + 1, 2) Then GoTo
ContinueUpdatingLowest

'Scramble LowestUsedElements with the same score by swapping

    'randomize

    R71 = Int((ScrambleLowestNumber * Rnd)) ' check this gives 0 to some number!!!
```

```
TempScrambleLowestAddress = LowestUsedElement(MarkScrambleLowestStart, 1)

TempScrambleLowestScore = LowestUsedElement(MarkScrambleLowestStart, 2)

LowestUsedElement(MarkScrambleLowestStart, 1) =
LowestUsedElement(MarkScrambleLowestStart + R71, 1)

LowestUsedElement(MarkScrambleLowestStart, 2) =
LowestUsedElement(MarkScrambleLowestStart + R71, 2)

LowestUsedElement(MarkScrambleLowestStart + R71, 1) = TempScrambleLowestAddress

LowestUsedElement(MarkScrambleLowestStart + R71, 2) = TempScrambleLowestScore

ScrambleLowestNumber = 0

MarkScrambleLowestStart = 0


End Select


ContinueUpdatingLowest:


Next I97



MissScramble:


End Sub


Public Sub PunishMutateNowSub()


If ActivatePunish = 0 Then GoTo MissOnPunishNows

'If RunningScoreTotal / RunningScoreWindow > 1 / 2 Then GoTo MissOnPunishNows

'If RunningScoreTotal >= RunningScoreWindow Then GoTo MissOnPunishNows

  'If DeltaRunningScore > 0 Then GoTo MissOnPunishNows


'Randomize

        'R26 = Int(RunningScoreWindow * Rnd)

        'If (R26 / RunningScoreWindow) < (RunningScoreTotal / RunningScoreWindow) Then GoTo
MissOnPunishNows
```

```
For ActRegLine = StartLoop To EndLoop

If ActRegLine = 0 Then GoTo MissActRegLineZero
PositionAR1 = 0
PositionNow1 = 0

For PositionAR1 = 1 To Anumber



    For PositionNow1 = 1 To Knumber
'randomize
MutationNow = Int((100 * Rnd) + 1)
If MutationNow < MutationThreshold Then GoTo NoPunishMutateNow
Sevencycle:
        'randomize
        R2 = Int((Enumber * Rnd) + 1)


'Avoid creating inputs at random
If R2 <= InputRange Then GoTo Sevencycle
'Avoid creating outputs at random
'If R2 = 10 Then GoTo Sevencycle 'Remove
'If R2 = 20 Then GoTo Sevencycle 'Remove
'Avoid selfing
If R2 = Activity(ActRegLine, PositionAR1) Then GoTo Sevencycle

Select Case Activity(ActRegLine, PositionAR1)

Case Is <= InputRange
'If R2 > Enumber / 2 Then GoTo Sevencycle
```

```
Case Is > Enumber - OutputRange
'If R2 < Enumber / 2 Then GoTo Sevencycle 'Remove

Case Else
'If (Activity(ActRegLine, PositionAR1)) Mod 2 < R2 Mod 2 Then GoTo Sevencycle
'If (Activity(ActRegLine, PositionAR1)) Mod 2 > R2 Mod 2 Then GoTo Sevencycle

End Select

Element(Activity(ActRegLine, PositionAR1), PositionNow1) = R2
NoPunishMutateNow:
            Next PositionNow1

    Next PositionAR1

MissActRegLineZero:
 Next ActRegLine

MissOnPunishNows:

End Sub
Public Sub PunishMutateNextSub()

If ActivatePunish = 0 Then GoTo MissOnPunishNexts
            'If RunningScoreTotal / RunningScoreWindow > 1 / 2 Then GoTo MissOnPunishNexts
'If RunningScoreTotal >= RunningScoreWindow Then GoTo MissOnPunishNexts
    'If DeltaRunningScore > 0 Then GoTo MissOnPunishNexts

If StartLoop = 0 Then StartLoop = 1
For ActRegLine = StartLoop To (EndLoop - 1)
PositionAR2 = 0
```

```
PositionNext1 = 0
For PositionAR2 = 1 To Anumber

        For PositionNext1 = 1 To Knumber
                MutationNext = Int((100 * Rnd) + 1)
If MutationNext < MutationThreshold Then GoTo NoPunishMutateNext

Eightcycle:
                'randomize
                R4 = Int((Enumber * Rnd) + 1)

                'no selfing
                If R4 = Activity(ActRegLine, PositionAR2) Then GoTo Eightcycle
                'no spurious input
                If R4 <= InputRange Then GoTo Eightcycle



                'Avoid creating outputs at random
'If R4 = 10 Then GoTo Eightcycle 'Remove
'If R4 = 20 Then GoTo Eightcycle 'Remove

Select Case Activity(ActRegLine, PositionAR2)

Case Is <= InputRange
'If R4 > Enumber / 2 Then GoTo Eightcycle

Case Is >= Enumber - OutputRange
'If R4 < Enumber / 2 Then GoTo Eightcycle 'Remove

Case Else
'If Activity(ActRegLine, PositionAR2) Mod 2 = R4 Mod 2 Then GoTo Eightcycle
```

```
End Select

Element(Activity(ActRegLine, PositionAR2), Knumber + PositionNext1) = R4

NoPunishMutateNext:
        Next PositionNext1

    Next PositionAR2
 Next ActRegLine

MissOnPunishNexts:

End Sub

Public Sub PunishMutateNextOverlap()


PositionAR2 = 0
PositionNext1 = 0
For PositionAR2 = 1 To Anumber

     If ActivatePunish = 0 Then GoTo MissOnPunishNextOverlaps
    'If RunningScoreTotal / RunningScoreWindow > 1 / 2 Then GoTo MissOnPunishNextOverlaps
      'If RunningScoreTotal >= RunningScoreWindow Then GoTo MissOnPunishNextOverlaps
    'If DeltaRunningScore > 0 Then GoTo MissOnPunishNextOverlaps

        For PositionNext1 = 1 To Knumber
                MutationNext = Int((100 * Rnd) + 1)
If MutationNext < MutationThreshold Then GoTo NoPunishMutateNextOverlap

Elevencycle:
                'randomize
```

```
            R4 = Int((Enumber * Rnd) + 1)

            'no selfing
            If R4 = Activity(StartLoop, PositionAR2) Then GoTo Elevencycle
            'no spurious input
            If R4 <= InputRange Then GoTo Elevencycle
            'Avoid creating outputs at random
'If R4 = 10 Then GoTo Elevencycle 'Remove
'If R4 = 20 Then GoTo Elevencycle 'Remove

'Select Case Activity(StartLoop, PositionAR2)

'Case Is < 4
'If R4 > Enumber / 2 Then GoTo Elevencycle

'Case Is = 10
'If R4 < Enumber / 2 Then GoTo Elevencycle

'Case Is = 20
'If R4 < Enumber / 2 Then GoTo Elevencycle

'Case Else

'End Select

Element(Activity(StartLoop, PositionAR2), Knumber + PositionNext1) = R4

NoPunishMutateNextOverlap:
      Next PositionNext1

   Next PositionAR2
```

```
MissOnPunishNextOverlaps:
End Sub



Public Sub RemoveSelfingSub()

'eliminates self-referencing
320                                             ' Repère 320
  I19 = 0
  J19 = 0
  For I19 = 1 To Enumber
    For J19 = 1 To Knumber * 2
ReplaceCycle:
      'randomize
      R1 = Int((Enumber * Rnd) + 1)
      If I19 = Element(I19, J19) Then Element(I19, J19) = R1
      If I19 = R1 Then GoTo ReplaceCycle
    Next J19
  Next I19

End Sub

Public Sub RemoveInputGenerationByCocoSub()
 'Eliminates a second input coming from Coco
'If there is a real input, it is in the first position in the AR
'so check the New line and the following lines for a second input and replace it at random
'this sub MUST be disabled if we want to run in an anticipatory mode in which inputs are predicted

I45 = 0
J45 = 0
InputTally = 0
```

```
RandomizeAgain = 0

Select Case NewLineNumber

Case Is = InputLine

    For I45 = 1 To Anumber
If Activity(NewLineNumber, I45) > 3 Then GoTo DontDoAnything1

    InputTally = InputTally + 1
    If InputTally < 2 Then GoTo DontDoAnything1
ReplaceInput1:
    'randomize
    RandomizeAgain = 0
    R16 = Int((Enumber * Rnd) + 1)
    If R16 < 4 Then RandomizeAgain = 1
    If R16 > Enumber - OutputRange Then RandomizeAgain = 1
    If RandomizeAgain = 1 Then GoTo ReplaceInput1
        For J45 = 1 To Anumber
        If R16 = Activity(NewLineNumber, J45) Then RandomizeAgain = 1
        Next J45
    If RandomizeAgain = 1 Then GoTo ReplaceInput1
    Activity(NewLineNumber, I45) = R16
    InputTally = InputTally - 1
'Victor addition to Coco 19 atelier 4-6-2008
DontDoAnything1:
    Next I45

Case Else

    For I45 = 1 To Anumber
If Activity(NewLineNumber, I45) > 3 Then GoTo DontDoAnything2
```

```
If Activity(NewLineNumber, I45) = Activity(InputLine, 1) Then GoTo DontDoAnything2 'allows the same input to be repeated
    InputTally = InputTally + 1
    If InputTally < 1 Then GoTo DontDoAnything2
ReplaceInput2:
    'randomize
    RandomizeAgain = 0
    R16 = Int((Enumber * Rnd) + 1)
    If R16 < 4 Then RandomizeAgain = 1
    If R16 > Enumber - OutputRange Then RandomizeAgain = 1
    If RandomizeAgain = 1 Then GoTo ReplaceInput2
        For J45 = 1 To Anumber
        If R16 = Activity(NewLineNumber, J45) Then RandomizeAgain = 1
        Next J45
    If RandomizeAgain = 1 Then GoTo ReplaceInput2
    Activity(NewLineNumber, I45) = R16
    InputTally = InputTally - 1
'Victor addition to Coco 19 atelier 4-6-2008
DontDoAnything2:
    Next I45


    End Select



End Sub
Public Sub RemoveSpuriousInputSub()
'Eliminates inputs in all fields so the system cannot learn!
330                                            ' Repère 330
    I20 = 0
    J20 = 0
    For I20 = 1 To Enumber
        For J20 = 1 To Knumber * 2
            If Element(I20, J20) > 3 Then GoTo ThisIsNotAnInput
```

```
ReplaceBiCycle:
            'randomize
            R3 = Int((Enumber * Rnd) + 1)
            If R3 < 4 Then GoTo ReplaceBiCycle
            Element(I20, J20) = R3
ThisIsNotAnInput:
        Next J20
    Next I20
'
End Sub
```

```
'
' %%%%%%%%%%%%%%%%%%%%%%%%%%%%%%%% MAURICE 05-06-2008 - PAQUET DES SÉQUENCES MAURICE %%%%%%%%%%%%%%%%%%%%%%%%%%%%%%%%%%%%%%%%%%%%%%%%%%%%%%%%%%%%
'Sub Patch()        ' REMPLACER TOUT LE PAQUET MAURICE PAR CELUI CI-DESSOUS ET SUPPRIMER CETTE LIGNE Sub
'
' %%%%%%%%%%%%%%%%%%%%%%%%%%%%%%%% MAURICE 05-06-2008 - PAQUET DES SÉQUENCES MAURICE %%%%%%%%%%%%%%%%%%%%%%%%%%%%%%%%%%%%%%%%%%%%%%%%%%%%%%%%%%%%
'
'
'
'%%%%%%%%%%%%%%%%%%%%%%%%%%%%%  Maurice 05-06-2008 - Début affichage tableau Elément sur Pause  %%%%%%%%%%%%%%%%%%%%%%%%%%%%%%%%%%%%%%%%%%%%%%%%%%%%
Public Sub Maurice_AffTbÉlémPause()                                    '%
'%
                    ' Maurice 03-06-2008 - Espionnage                     '%
```

```
600                                 ' Repère 600 sur lequel on revient si l'option Pause n'est pas active
'%
   DoEvents                                                                     '%
                                                                        '%
'
%%%%%%%%%%%%%%%%%%%%%%%%%%%%%%%%%%%%%%%%%%%%%%%%%%%%%%%%%%%%%%%%%%%%%%%%%%%%%%%%%%%%%%%%%%%%%%%%%%%%%%%%%%%%%%%%%%%%%%%%%%%%%%%%%%%%%%%%%%%%%%%%%%%%%%%%%%%%%%%%%%%%%%%%%%%
601
                                                                          '%
End Sub                                                                        '%



'
'%%%%%%%%%%%%%%%%%%%%%%%%%%%%%%%%%%%%%%%%%%%%%%%%%%%%%  Maurice - 05-06-2008 - Début des affichages
en fin de programme  %%%%%%%%%%%%%%%%%%%%%%%%%%%%%%%%%%%%%%%%%%%%%%%%%%%%%%%%%%%%%%%%%%%%%%%%%%%%%%%%
'                                                                            '%
Public Sub Maurice_AffTbÉlémFinProg()       '                                              '%
'%
   If Option1.Value = True Then GoTo Affiche_Élémentsf         '          - id -
'%
   If Option2.Value = True Then GoTo Affiche_LongTermMemorySubf    '          - id -
'%
'                                                                            '%
'          1) - Tableau des éléments                                                '%
Affiche_Élémentsf:                                                                  '%
   Dim xt, yt, i, j As Integer                                                      '%
   Cls                                                                         '%
   CurrentX = 3100: CurrentY = 6410                                                      '%
   Print " Now                                            "                   '%
   CurrentX = 3100: CurrentY = 6410                                                      '%
   ForeColor = RGB(255, 0, 0)                                                          '%
   Print "                                                Next"            '%
   ForeColor = RGB(0, 0, 0)                                                           '%
   xt = 1000: yt = 6660                                                              '%
```

```
   For i = 1 To Enumber                              ' Attention si Enumber > 32, dépassement capacité
'%
      CurrentX = 900: CurrentY = yt                                                    '%
      ForeColor = RGB(0, 0, 255)                                                       '%
      Print i                                                                          '%
      ForeColor = RGB(0, 0, 0)                                                         '%
      For j = 1 To 2 * Knumber                        ' Attention si Knumber > 10, dépassement
capacité          '%
         If j = Knumber + 1 Then xt = xt + 800 Else xt = xt + 400  '                  - id -
'%
         If j > Knumber Then ForeColor = RGB(255, 0, 0)            '                  - id -
'%
         CurrentX = xt: CurrentY = yt                                                  '%
         Print Element(i, j)                                                           '%
      Next j                                                                           '%
   ForeColor = RGB(0, 0, 0)                                                            '%
   xt = 1000: yt = yt + 245                                                            '%
   Next i                                                                              '%
'%
   GoTo Fin_Affichage_Fin_Prog                                                         '%
'                                                                                      '%
'          2) - Table Activity Long Term                                               '%
'                                                                                      '%
Affiche_LongTermMemorySubf:                                                            '%
   Dim Tltmf, Hltmf, Vltmf, Xltmf, Yltmf As Integer
'%
   Cls                                                                                 '%
   Form1.Font.Size = 7                                                                 '%
   CurrentX = 150: Xltmf = 150                                                         '%
   CurrentY = 6450: Yltmf = 6450                                                       '%
   ForeColor = RGB(0, 0, 255)                                                          '%
   For Tltmf = 1 To Enumber                                                            '%
      Xltmf = Xltmf + 580: CurrentX = Xltmf: CurrentY = Yltmf
'%
      Print Tltmf                                                                      '%
```

```
    Next Tltmf                                                          '%
'                                                                       '%
    For Hltmf = 1 To Enumber                                            '%
        Yltmf = Yltmf + 240                                             '%
        CurrentX = 150: Xltmf = 150                                     '%
        CurrentY = Yltmf                                                '%
        ForeColor = RGB(0, 0, 255)                                      '%
        Print Hltmf                                                     '%
        ForeColor = RGB(0, 0, 0)                                        '%
        For Vltmf = 1 To Enumber                                        '%
            Xltmf = Xltmf + 580: CurrentX = Xltmf                       '%
            CurrentY = Yltmf                                            '%
            Print LTMemory(Hltmf, Vltmf)                                '%
        Next Vltmf                                                      '%
     Next Hltmf                                                         '%
'                                                                       '%
Fin_Affichage_Fin_Prog:                       '                         '%
End Sub                                                                 '%
'                                                                       '%
'%%%%%%%%%%%%%%%%%%%%%%%%%%%%%%%%  Maurice - 05-06-2008 - Fin des affichages
en fin de programme  %%%%%%%%%%%%%%%%%%%%%%%%%%%%%%%%%%%%%%%%%%%%
'



'
' %%%%%%%%%%%%%%%%%%%%%%%%%%%%%%%%%%%%%%%%%% Maurice - 05-06-2008 -
Début Calcul Table Activity
%%%%%%%%%%%%%%%%%%%%%%%%%%%%%%%%%%%%%%%%%%%%%%
Private Sub Maurice_AffTbActivity()              ' %%%% Maurice - 03-06-2008 - affichage du tableau
Activity                  '%
'                                                                       '%
' En entrée Table Activity indexée par la ligne la + récente NewLineNumber et le n° de colonne k = 1
à Anumber                 '%
```

```
'	Le nb de lignes de la table Activity est paramétré par EndOfActivityRegister (150 à ce jour)
'%
' En sortie Tableau 'Activity Register Line' sur feuille principale
'%
'	La ligne la + récente de l'entrée sera mise en fin du tableau 'Activity Register Line' (ligne 10)
'%
'	On calcule les n° de ligne de la table Activity à positionner en ligne (Ln) du tableau 'Activity
Register Line' :   '%
'		Si NewLineNumber > 9      alors Ltn = NewLineNumber - 9
'%
'		Si NewLineNumber < ou = 9 alors Ltn = EndOfActivityRegister + NewLineNumber - 9
'%
'                                                                        '%
' Positionnement des n° de ligne de la table Activity                                  '%
  If NewLineNumber > 12 Then                                                         '%
    Ln(0) = NewLineNumber - 12                                                       '%
  Else                                                                  '%
    Ln(0) = EndOfActivityRegister + NewLineNumber - 12
'%
  End If                                                                '%
  For I13 = 1 To 12                                                          '%
    If Ln(I13 - 1) = EndOfActivityRegister Then                                         '%
      Ln(I13) = 1                                                         '%
    Else                                                                '%
      Ln(I13) = Ln(I13 - 1) + 1                                                  '%
    End If                                                              '%
  Next I13                                                               '%
                                                                  '%
' Positionnement des valeurs de la table Activity dans le champ          '%
' 'Activity Register Line' affiché à l'écran                           '%
'                                                            '%
Dim ColMax As Integer
ColMax = 13

Select Case ColMax '%
```

```
Case Is < Anumber
    For I13 = 1 To 12 'number of rows to display                                    '%
        For I14 = 1 To ColMax
        LActivity(((I13 - 1) * 13) + (I14 - 1)) = Activity(Ln(I13 - 1), I14)
        Next I14
    Next I13

Case Is = Anumber
    For I13 = 1 To 12 'number of rows to display                                    '%
        For I14 = 1 To ColMax                                   '%
            LActivity(((I13 - 1) * 13) + (I14 - 1)) = Activity(Ln(I13 - 1), I14)
        Next I14                                                '%
    Next I13

Case Is > Anumber 'so don't display those values in the ActivityRegister in positions > 13 (e.g., 14, 15
etc.)
    For I13 = 1 To 12 'number of rows to display                                    '%
        For I14 = (Anumber + 1) To ColMax
        LActivity(((I13 - 1) * 13) + (I14 - 1)) = 0
        Next I14
    Next I13
    For I13 = 1 To 12 'number of rows to display                                    '%
        For I14 = 1 To Anumber
        LActivity(((I13 - 1) * 13) + (I14 - 1)) = Activity(Ln(I13 - 1), I14)
        Next I14
    Next I13

Case Else

                                '%

  End Select
```

'%

' %%%%%%%%%%%%%%%%%%%%%%%%%%%%%%%%%%%%%% Maurice - 05-06-2008 - Fin Calcul Table Activity %%

'

'

'

%% FIN DES PAQUETS MAURICE %%%

'

End Sub
